# Network analysis of EMT and MET micro-RNA regulation in breast cancer

**DOI:** 10.1038/s41598-017-13903-1

**Published:** 2017-10-19

**Authors:** Diana Drago-García, Jesús Espinal-Enríquez, Enrique Hernández-Lemus

**Affiliations:** 1 Computational Genomics Division, National Institute of Genomic Medicine (INMEGEN), Mexico City, 14610 Mexico; 2 Centro de Ciencias de la Complejidad, Universidad Nacional Autónoma de México (UNAM), Mexico, 04510 Mexico

## Abstract

Over the last years, microRNAs (miRs) have shown to be crucial for breast tumour establishment and progression. To understand the influence that miRs have over transcriptional regulation in breast cancer, we constructed mutual information networks from 86 TCGA matched breast invasive carcinoma and control tissue RNA-Seq and miRNA-Seq sequencing data. We show that miRs are determinant for tumour and control data network structure. In tumour data network, miR-200, miR-199 and neighbour miRs seem to cooperate on the regulation of the acquisition of epithelial and mesenchymal traits by the biological processes: Epithelial-Mesenchymal Transition (EMT) and Mesenchymal to Epithelial Transition (MET). Despite structural differences between tumour and control networks, we found a conserved set of associations between miR-200 family members and genes such as VIM, ZEB-1/2 and TWIST-1/2. Further, a large number of miRs observed in tumour network mapped to a specific chromosomal location in DLK1-DIO3 (Chr14q32); some of those miRs have also been associated with EMT and MET regulation. Pathways related to EMT and TGF-beta reinforce the relevance of miR-200, miR-199 and DLK1-DIO3 cluster in breast cancer. With this approach, we stress that miR inclusion in gene regulatory network construction improves our understanding of the regulatory mechanisms underlying breast cancer biology.

## Introduction

Breast cancer is the most frequent cancer among women, and the second most common cancer in the world^1^. The high incidence, mortality, and clinical heterogeneity highlights the urgency for a better understanding of breast cancer development. Over the last years, with the introduction of next generation sequencing technologies, a group of regulatory small non-coding RNA molecules known as microRNAs (miRs) have shown to be crucial for breast tumour establishment and progression (reviewed in^2^).

miR activity has been associated with the transcriptional regulation of many cellular mechanisms, including those involved in cancer, for instance: apoptosis, proliferation, and migration^3^. These miRs who may act as “oncogenes” or “tumour suppressor genes” are referred altogether as “oncomiRs” (reviewed in^4^). Specific miR regulation and co-regulation mechanisms over genes and other miRs are associated with cancer mechanisms^5^, and some have been specifically linked to breast cancer biology and prognosis^2^.

There is evidence that miR activity impacts in a robust manner over protein levels, mainly through mRNA destabilization^6^, making transcriptome profiling technologies especially useful for miR regulation analysis. Mechanisms involved in miR regulation act through direct and indirect interactions^7^, possibly favouring target upregulation or downregulation^8^. Regulatory relationships between miR and genes (including transcription factors), are known to participate in mechanisms that ensure biological robustness^9^, and to produce co-expression profiles that are determinant of the phenotype^10^.

To uncover the regulatory relationship between miRs and genes (mRNAs), several computational approaches have been developed^11–17^. Recent efforts are focused on the integration of miR-target databases and miR-mRNA expression profiles. These tools measure the relationship between miR and mRNA expression profiles, mainly relying on linear correlation^11,12^, or Bayesian models^18^. Acknowledging the non-linear nature of the majority of biological relationships, algorithms based on non-linear correlation have proven to accurately capture miR-mRNA regulatory associations^13–15^.

To understand the transcriptional relation between miRs and mRNAs we constructed networks with primary breast cancer and matched control tissue sequencing data from TCGA using Mutual Information (MI), a non-linear correlation measure. We show that miR-199 and miR-200 are determinant for the structure of the networks inferred from tumour and control data, respectively. Further, a large number of miRs observed in the network inferred from tumour data mapped to a large miR cluster in chromosome 14 (DLK1-DIO3 region). We found that miR-200, miR-199 and miRs mapping to DLK1-DIO3 cluster in the network inferred from tumour data seem to cooperate in the regulation of the acquisition of epithelial and mesenchymal traits in a set of processes described as Epithelial-Mesenchymal plasticity (EMP). Despite the structural differences between networks inferred from tumour and control data, we found a conserved core of nodes and edges associated to miR-200 family. This core is characterized by miR-200 family members overexpression, meanwhile Epithelial to Mesenchymal Transition (EMT) transcription factors and marker genes such as VIM, ZEB-1/2 and TWIST-1/2^19–21^ are underexpressed. This expression signature is related to epithelial trait acquisition process: Mesenchymal to Epithelial Transition (MET), which has proven to be important for tumour malignancy^22,23^. Finally, an analysis regarding pathways^24^ shows that EMT and TGF-beta pathways, crucial processes involved in breast cancer, are deregulated in our tumour samples for three different pathway databases: KEGG, Wikipathways and Reactome. We also assess the presence of relevant miRs in our inferred networks with validated or previously predicted miR-mRNA associations.

These results altogether show that miR regulation and their associated functions are important for cancer pathogenesis, specially miR-199 and miRs from DLK1-DIO3 cluster, whose involvement in breast cancer is still under study. The work presented here attempts to stress that the inclusion of these small RNAs in the network construction seems to be relevant to understand regulatory mechanisms underlying breast cancer biology.

## Results

We define network nodes as mRNA and miRs (mature miR); for most of our analyses the mentioned miRs are grouped in miR families. Inferred network edges are defined as the MI between a pair of nodes, resulting in an undirected network with three types of associations: between miRs (miR-miR), between mRNAs (mRNA-mRNA), and between miRs and mRNAs (miR-mRNA).

### The MI values inferred from tumour data are lower than in the control

**Figure 1:**
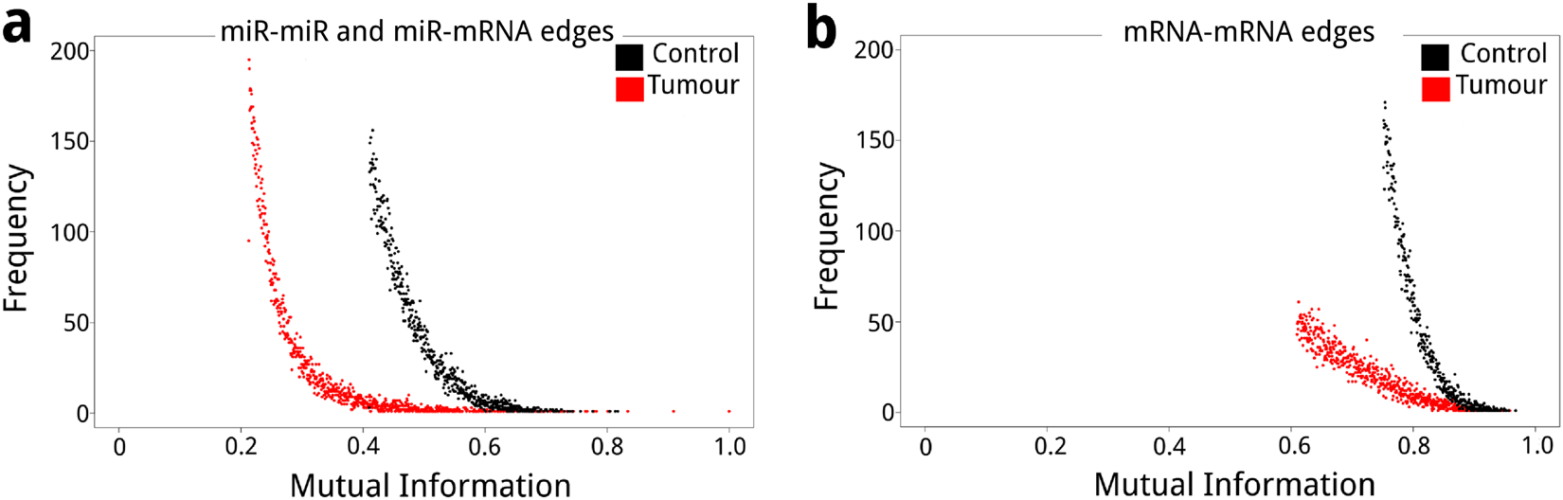
MI histograms for network edges; (**a**) overlayed miR-miR and miR-mRNA largest connected component edges for networks inferred from tumour (red) and control (black) data (0.259% strongest edges), and (**b**) mRNA-mRNA edges for the largest connected component edges for networks inferred from tumour (red) and control (black) data (0.013% strongest edges).

From non-cancerous adjacent breast tissue and coupled primary breast cancer tissue expression data we constructed mutual information-based networks using ARACNe^25^. We used two different cut-off values for miR-miR and miR-mRNA, and mRNA-mRNA edges (see Methods). As shown (Fig. 1), there were quantitative differences in the distribution of MI values between miRs and mRNAs, which may reflect the differences in the nature of the molecules. Further, the distribution of MI values from the tumour (red) and control (black) networks vary for miR-miR and miR-mRNA edges (Fig. 1a), and mRNA-mRNA edges (Fig. 1b), such that the lowest MI values for tumour edges are biased to the left; meanwhile at the same threshold, the lowest MI values for control edges tend to be higher. These differences are conserved on the whole set of MI edges (Supplementary Fig. S1).

### Topological properties of inferred networks

**Figure 2:**
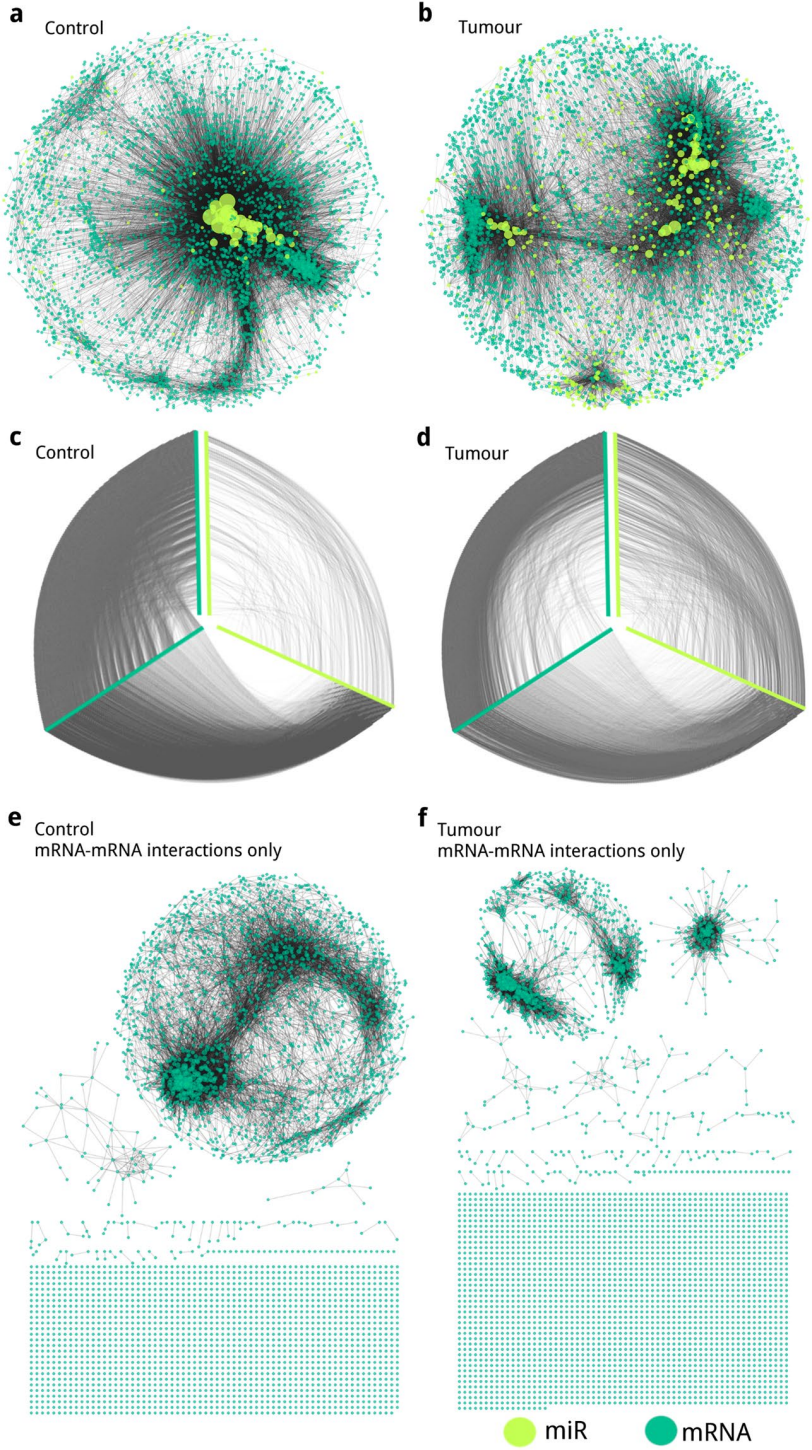
Representation of Large component networks inferred from (**a**) control and (**b**) tumour data. Hiveplot representation of the networks inferred from (**c**) control, and (**d**) tumour data. Yellow-green nodes represent miRs, meanwhile turquoise nodes represent mRNAs. For (**a**) and (**b**) node size is proportional to node degree. For (**c**) and (**d**) hiveplot visualization: network nodes are represented in the axes ordered by increasing degree from its centre; edges between yellow-green axes represent miR-miR edges; between turquoise axes the mRNA-mRNA edges; and between yellow-green and turquoise axes the miR-mRNA ones. Effect on component disintegration after mRNA removal from the largest component of the networks inferred from (**e**) control and (**f**) tumour data.

We analysed miR and mRNA contribution on network nodes and edges, resulting parameters are described in Supplementary Table S1. We decided to focus mainly on the largest connected component for each network (Fig. 2) since they conserve almost all nodes and edges. Whole networks inferred from tumour and control data can be found in Supplementary Figs S2 and S3, respectively.

The largest components of the networks inferred from tumour (Fig. 2b,d) and control (Fig. 2a,c) data have a similar number of nodes; although the frequency of nodes that correspond to miR in the network inferred from tumour data is close to fourfold larger compared to controls. Also, as may be observed in the hiveplot representations (Fig. 2c,d), there are less miR-mRNA and mRNA-mRNA edges in the network inferred from tumour data, and the number of miR-miR edges in the tumour data network are over sevenfold larger compared to the control data one (Supplementary Table S1).

### miRs maintain network cohesion

If we only observe resulting networks by their mRNA-mRNA component and remove all miRs and their edges, the number of components increases since thousands of single nodes appear. This behaviour can be observed in Fig. 2e,f and Supplementary Table S1 ‘Large component mRNA-mRNA subnetwork’ section, indicating that miRs are important for network cohesion.

### Networks inferred from tumour and control data show different biological enrichment

To study the networks’ biological significance we performed a Gene Ontology (GO) enrichment analysis. We included annotations for three aspects used to the describe the associated mRNAs function: biological process, molecular function and cellular component categories.

The GO enrichment for controls and tumours largest connected component network mRNAs showed important differences in the processes, functions, and components enriched for each network. Even though the number of input mRNAs is similar for tumours and controls, we obtained 128 enriched GO terms for controls versus 446 for tumours. The top five most significant GO terms that covered our selected criteria (see Methods) for tumour and control samples can be found in Supplementary Table S2; we found that the results for the control analysis are mainly related to translation, transcription, and signal transduction. Meanwhile, tumour enrichment analysis shows processes related to immune response, and cell adhesion; functions related to extracellular molecule binding along with extracellular space and adhesion cellular components. The entire results can be found online as Supplementary Tables S3 and S4 for control and tumour data, respectively.

### miR-200 and miR-199 define networks structure and function

Besides the larger frequency of miR and miR-miR edges for tumours, there are important differences in node degree between the networks inferred from tumour and control data. Top ten degree nodes for each network were selected (Supplementary Table S5), and we found they were only miR for controls and tumours. Also, it can be observed that controls top degree nodes are consistently higher compared to tumours.

### Top degree nodes result in miR-200 and miR-199

To study the biological role of this highly connected miR we decided to analyse them as miR families and networks resulting from nodes that are directly connected to them (first neighbours). Instead of focusing on individual mature miR we started our analysis focusing on miR families. miR families have proven to be advantageous to group miR genes, due to their predictive power based in the miR family members structural similitude^26^. We focused on miR-200 and miR-199 since most of their members are on the top highly connected nodes for controls and tumours respectively.

### miR-200 is relevant for networks inferred from tumour and control data

**Figure 3:**
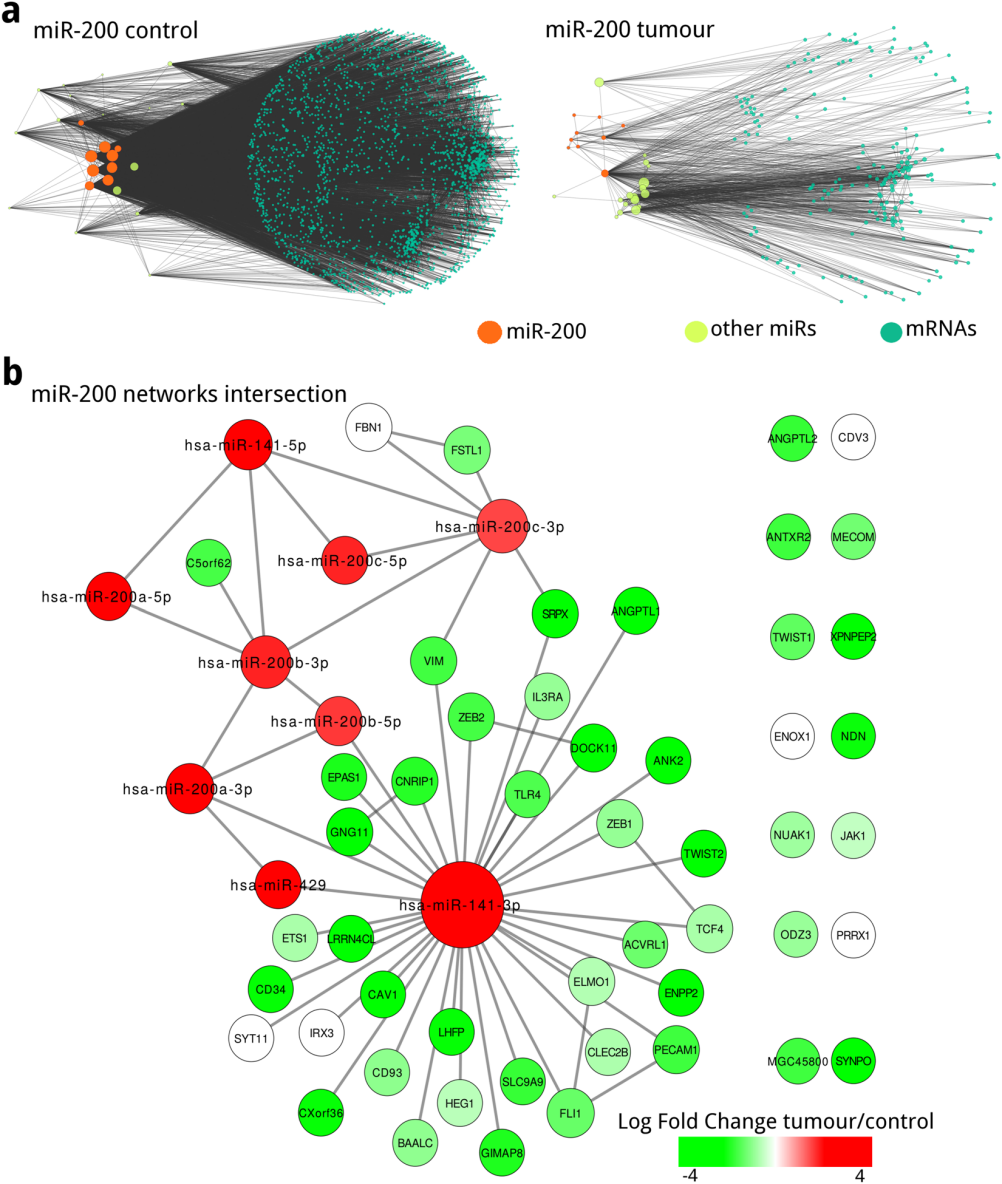
miR-200 first neighbours of the sub-networks inferred from (**a**) control and (**b**) tumour data; node sizes correspond to node degree. (**c**) Intersection of the miR-200 networks inferred from control and tumour data, conserved nodes and edges are coloured according to their differential expression. Notice that miRs are overexpressed meanwhile the majority of mRNAs are underexpressed.

Top degree miR family for controls is miR-200, constituted by: hsa-miR-200a-5p, hsa-miR-200a-3p, hsa-miR-200b-5p, hsa-miR-200b-3p, hsa-miR-200c-5p, hsa-miR-200c-3p, hsa-miR-141-5p, hsa-miR-141-3p, and hsa-miR-429; all of them were present in the largest connected component networks inferred from control and tumour data. We constructed networks with miR-200 first neighbours (miR and mRNA), and obtained a network inferred from control data with 2,272 nodes and 16,923 edges (Fig. 3a) and a network inferred from tumour data with 224 nodes with 1,046 edges (Fig. 3b). As expected, miR-200 degrees are larger in the network inferred from control data compared to the network inferred from tumour data (Supplementary Table S6). The network inferred from control data has more interacting mRNAs, and more edges between the mRNAs and miR-200 (2,247 mRNAs for controls and 198 for tumours). Although there is almost no difference in the number of miRs between the miR-200 networks for control and tumour data (25 miR for controls and 26 miR for tumours).

Enrichment analysis of the mRNAs present in the tumour data miR-200 first neighbours network showed different processes compared to controls. Even if miR-200 is present in both networks the targets to their regulation are different, as we obtained 33 GO terms for controls enrichment analysis versus the 62 obtained for tumours, even though input mRNAs were 2,247 for controls and 198 for tumours (The entire analysis results can be found online as Supplementary Tables S7 and S8 for control and tumour data, respectively). These differences between miR-200 enrichment are evident at the top 5 most significant processes, functions and components depicted in Table 1.

**Table 1:** Enrichment analysis for miR-200 networks inferred from control and tumour data.

miR-200 miR family Enrichment			
**Control**		**Tumour**	
	FDR		FDR
**Biological process**		**Biological process**	
Cell adhesion	$2.8480$ × ${10}^{-4}$	Regulation of response to stimulus	$1.6702$ × ${10}^{-6}$
Biological adhesion	$2.9344$ × ${10}^{-4}$	Regulation of developmental process	$1.6702$ × ${10}^{-6}$
		Cellular component movement	$3.3965$ × ${10}^{-6}$
		Regulation of cell proliferation	$3.3965$ × ${10}^{-6}$
		Regulation of immune system process	$9.4147$ × ${10}^{-6}$
**Molecular function**		**Molecular function**	
Transmembrane receptor protein	$3.6305$ × ${10}^{-5}$	Receptor binding	$1.9975$ × ${10}^{-6}$
kinase activity			
Transmembrane receptor protein	$9.7399$ × ${10}^{-5}$	Carbohydrate binding	$1.2562$ × ${10}^{-5}$
tyrosine kinase activity			
Protein tyrosine kinase activity	$3.6087$ × ${10}^{-3}$	Polysaccharide binding	$6.1321$ × ${10}^{-4}$
Protein kinase activity	$1.0257$ × ${10}^{-2}$	Pattern binding	$6.1321$ × ${10}^{-4}$
		Growth factor binding	$9.2541$ × ${10}^{-4}$
**Cellular component**		**Cellular component**	
Cell junction	$8.6796$ × ${10}^{-13}$	E×tracellular region part	$9.1753$ × ${10}^{-9}$
Anchoring junction	$6.3052$ × ${10}^{-11}$	E×tracellular space	$9.4147$ × ${10}^{-6}$
Cell-cell junction	$6.3052$ × ${10}^{-11}$	E×tracellular matrix	$3.3656$ × ${10}^{-4}$
Adherens junction	$1.0953$ × ${10}^{-7}$	Cell surface	$3.3656$ × ${10}^{-4}$
Basolateral plasma membrane	$1.0953$ × ${10}^{-7}$	Proteinaceous extracellular matrix	$5.0306$ × ${10}^{-4}$
**miR-199 Enrichment**			
**Tumour**		FDR	
**Biological process**			
Biological adhesion		$8.5209$ × ${10}^{-29}$	
Cell adhesion		$8.5209$ × ${10}^{-29}$	
Extracellular structure organization		$1.0724$ × ${10}^{-13}$	
Skeletal system development		$3.9744$ × ${10}^{-13}$	
Extracellular matrix organization		$1.9525$ × ${10}^{-12}$	
**Molecular function**			
Calcium ion binding		$1.1340$ × ${10}^{-16}$	
Extracellular matrix structural constituent		$2.8499$ × ${10}^{-11}$	
Integrin binding		$7.4108$ × ${10}^{-9}$	
Glycosaminoglycan binding		$1.0312$ × ${10}^{-6}$	
Pattern binding		$5.5217$ × ${10}^{-6}$	
**Cellular component**			
Extracellular matrix		$4.0521$ × ${10}^{-42}$	
Proteinaceous extracellular matrix		$7.9803$ × ${10}^{-41}$	
Extracellular region part		$1.2162$ × ${10}^{-27}$	
Calcium ion binding		$1.1340$ × ${10}^{-16}$	
Extracellular matrix part		$1.8404$ × ${10}^{-13}$	

### miR-200 networks intersection show a common core related to EMT and MET

Even if miR-200 networks differ greatly in neighbour count and connectivity there are 59 common mRNAs between tumours and controls maintaining the same edges. Among those common mRNAs we can observe VIM, ZEB-1/2 and TWIST-1/2; mRNAs that together with miR-200 are associated to EMT/MET^19–21^. It should be noted that even if the edges between miR-200 and mRNAs are conserved in both networks their expression values between tumours and controls differs greatly, shifting abruptly; with miR-200 being highly overexpressed and neighbouring interacting mRNAs being highly underexpressed, as it can be observed in Fig. 3c.

### miR-199 behaviour is determinant for the network inferred from tumour data structure

**Figure 4:**
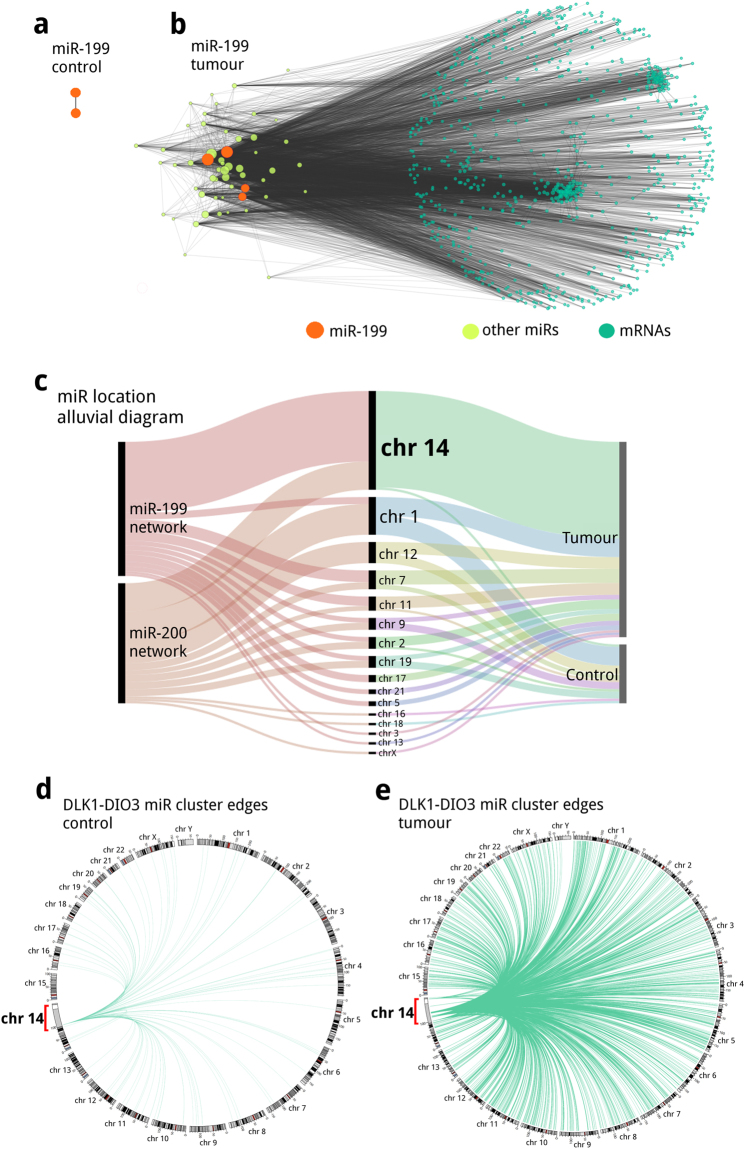
miR-199 first neighbours networks for (**a**) controls and (**b**) tumours; node sizes correspond to node degree. (**c**) Alluvial diagram of the chromosomal location of miRs present in miR-200 and miR-199 first neighbours networks. Circos plot representing the miRs in DLK1-DIO3 cluster (highlighted in red and amplified 100x) direct edges (miR-miR and miR-mRNA) from the Large component (**d)** controls and (**e**) tumours networks.

Top degree miR family for tumours network is miR-199, constituted by: hsa-miR-199b-5p, hsa-miR-199b-3p, hsa-miR-199a-5p and hsa-miR-199a-3p. The miR-199 miRs are present in the largest connected component of the network inferred from tumour data, in the network from control data only two members were present as an independent two node network (degree = 1) (Fig. 4a). Selecting miR-199 and their first neighbours (miR and mRNA) we obtained a network with 834 nodes and 7,053 edges (Fig. 4b) for tumour data. In agreement with the presented results, miR-199 degrees on the network inferred from tumour data (Supplementary Table S9) are smaller than miR-200 degrees for controls, even if they are the most connected miR family of their respective networks.

The mRNAs in miR-199 first neighbour network enriched to 119 GO terms (The entire analysis results can be found online at Supplementary Table S10). The top 5 most significant processes (Table 1) were mainly related to cellular adhesion, extracellular organization and development; enriched functions include molecule binding along with extracellular matrix involvement; and enriched cellular components are also centred on extracellular matrix. Controls network contains no mRNAs, so there are no enriched GO terms. It is worth mentioning that enriched GO-terms are similar to those found in the miR-200 tumour data network.

### The network inferred from tumour data miRs is locally enriched at the DLK1-DIO3 cluster

Examining miR-200 and miR-199 first neighbour networks from control data, miR-200 members appear to interact with a restricted number of miRs located in different chromosomes, with no obvious preference or pattern. In contrast, within the network inferred from tumour data, miRs show a local enrichment on chromosome 14 (Fig. 4c).

The chromosomal location of miRs mapping to chromosome 14 shows that they are located in a well defined region known as the DLK1-DIO3 cluster. This region in chr14.q32 is characterized for having a high density of miRs and other long non-coding genes. A circos plot in Fig. 4 shows the miRs mapping to the DLK1-DIO3 cluster direct edges (from the Large component networks) in the network inferred from tumour data (Fig. 4e) and the network inferred from control data (Fig. 4d). The tumour data network exhibits a larger number of cluster associations to multiple locations in the genome (Fig. 4e). As it can be observed in Fig. 4d,e there are a few associations involving the DLK1-DIO3 miRs and chromosome Y; these interactions arise from our data set including a male sample (TCGA-A0DD). The miR from DLK1-DIO3 cluster that are present in the tumour data network are mainly underexpressed (Supplementary Fig. S4).

### Pathway Analysis: miRs and deregulation of EMT pathways

We selected the Reactome, WikiPathways, and KEGG pathways which contained at least one gene that corresponds to mRNA nodes in the miR-200 and DLK1-DIO3 first neighbour networks inferred from tumour data (see Methods). Using Pathifier^24^, we estimated the Pathway Deregulation Score (PDS) of each sample and for the selected pathways^24^. We used Pathifier as it allows to integrate the mRNA expression and pathway information from each sample in a context-specific metric (PDS) that reflects pathway alterations according to the control behaviour in our dataset^24^.

We obtained a PDS matrix for 393 Reactome pathways (Supplementary Fig. S5 and Table S11), 237 for WikiPathways (Supplementary Fig. S6 and Table S12), and 133 for KEGG databases (Supplementary Fig. S7 and Table S13) containing the DLK1-DIO3 mRNA nodes. In the case of miR-200 mRNA nodes we obtained a PDS for 193 pathways in Reactome (Supplementary Fig. S8 and Table S14), 159 for WikiPathways (Supplementary Fig. S9 and Table S15), and 79 for KEGG databases (Supplementary Fig. S10 and Table S16).

**Figure 5:**
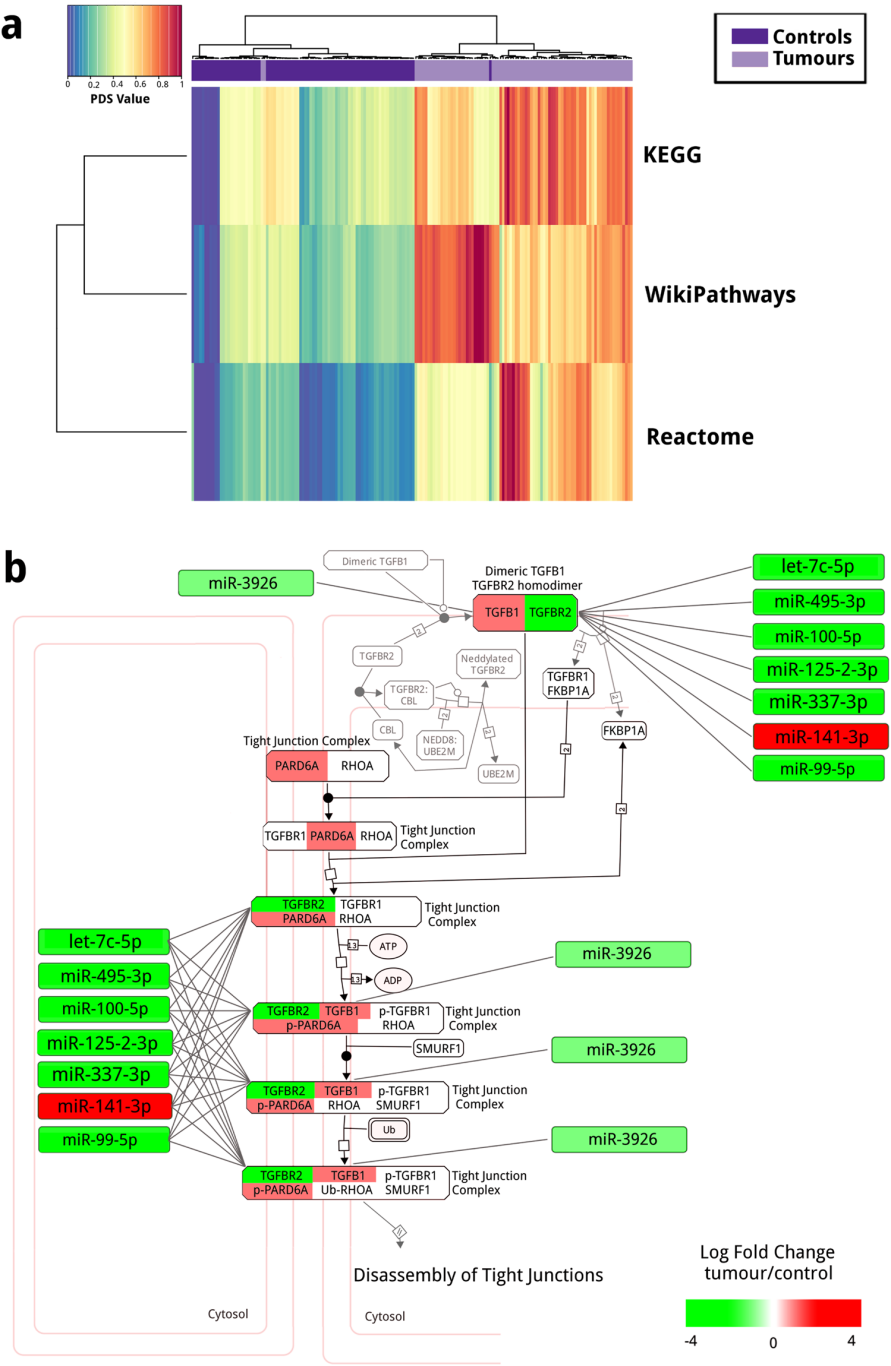
Pathway Analysis. (**a**) Pathway Deregulation Score heatmap for EMT related pathways from Reactome, WikiPathways and KEGG. (**b**) miRs from the network inferred from tumour data have associations with genes in the pathway (Nodes are coloured according to their expression).

We searched for common pathways between the miR-200 and the DKL1-DIO3 analyses, and found deregulated EMT-related pathways in each database. These pathways correspond to the KEGG TGF-beta signalling pathway, the Reactome TGF-beta receptor signalling in EMT (epithelial to mesenchymal transition) pathway, and the WikiPathways TGF-beta signalling in thyroid cells for epithelial-mesenchymal transition pathway (Fig. 5a). Specifically for the Reactome pathway, Fig. 5b shows the regulatory relationship between hsa-miR-141-3p and TGFBR2, as well as DLK1-DIO3 miRs involvement.

By looking at the first neighbours of the pathway genes corresponding to mRNAs in our networks inferred from tumour data, we found that several miR-200 and DLK1-DIO3 cluster miRs share associations with genes participating in those pathways (Supplementary Figs S11, S12 and S13). It is worth noticing that hsa-miR-141-3p consistently appears in the EMT pathways for the different databases.

### Network miRs are consistent with tumour phenotype

Networks inferred from tumour data have many miRs with reported functions related to tumour promotion, some of them are involved in breast cancer (Table 2), however, those functions are consistent with their expression profiles.

**Table 2:** Relevant miRs obtained by network analysis and their involvement in cancer. This table shows miRs first neighbours of miR-200 or miR-199, and miRs in DLK1-DIO3 cluster.

miR	Involvement in cancer	Expression		Reference
	miR-200 tumour network	−5p	−3p	
miR-381	Indirect supressor of migration,		−1.49	^83^
	associated to self-renewal			^84^
miR-379	Underexpressed in breast cancer,	−1.19	−1.40	^85^
	cyclin B1 was identified as target			
miR-100	Supression of breast cancer cells migration	−1.69		^86^
	though inhibition of Wnt/beta-catenin pathway			
	EMT activator, tumorigenesis and invasion inhibitor			^86^
miR-96	Strongly upregulated in breast cancer, important for	3.16		^87^
	cell growth and migration			
**miR-199 tumour network**		**5-p**	**3-p**	
miR-145	Inhibit growth and migration of breast cancer cells	−2.27	−1.52	^88^
miR-656	Downregulated in multiple human cancers,		−1.29	^89^
	lining it with tumour suppression			
miR-655	Linked to MET inhibition in breast cancer, upregulation is		−1.01	^90^
	linked to inhibition of migration and invasion			
miR-493	Reduced survival of breast cancer patients with aggressive			^91^
	tumours and microtubule drugs resistance	1.35	1.49	
**DLK1-DIO3 cluster**		**5-p**	**3-p**	
miR-379	Related to EMT and EMT in a prostate cancer model	−1.19	−1.40	^92^
miR-495	Linked to repression of the signalling between TWIST-1, SMI-1, ZEB-1/2		−1.50	^52^
	and miR-200			

### miR edges match associations in miRTarBase and TargetScan

We used the available information from the experimentally validated interactions in miRTarBase^27^ and the miR-target prediction associations from TargetScan^28^, to assess the presence of common interactions between the databases and our inferred networks (Supplementary Table S17). From our miR-200 first neighbour network inferred from tumour data, we evaluated the nodes and edges that mapped to a validated or predicted miR-mRNA association (Supplementary Table S18 and Supplementary Fig. S14). Again, in these *merged networks*, hsa-miR-141-3p appears consistently.

### miR-mRNA inference comparison

We compared the associations inferred by our proposed methodology with the miR-mRNA prediction results obtained by miRTarVis^15^ and our data. We used the miRTarVis implementation of algorithms that predict miR-mRNA associations by their expression profiles, relying on linear and non-linear correlation measures, as well as a Bayesian model^15^. Altogether, we obtained 757 matching edges out of 4,186 DLK1-DIO3 miR-mRNA associations (Supplementary Table S19), and 403 out of 1,535 miR-199-mRNA edges (Supplementary Table S20).

## Discussion

To understand the relationship between miRs and mRNAs in breast cancer, using paired RNA-Seq and miRNA-Seq data from 86 invasive breast cancer patients, we constructed and analysed gene regulatory networks from tumour and control data. These networks were inferred by means of an information theoretic approach, where nodes correspond to miRs and mRNAs, and their edges are the statistical dependence (MI) between their expression across samples. Resulting networks showed differences in their MI distributions and their structure. The comparison of the MI distribution between the networks inferred from tumour and control data in Fig. 1a,b shows that tumour edges tend to have lower MI values, and topological differences observed in Fig. 2 are evident. In the constructed networks, we noticed a miR cohesive property as shown in Fig. 2e,f; and top degree nodes correspondence to miR families: miR-200 for the control data network (Fig. 3a,b) and miR-199 for the tumour data network (Fig. 4a,b). Our miR family first neighbour networks inferred from tumour and control showed differential enrichment analysis results (Table 1), although miR-200 networks shared a common core (Fig. 3c). Regarding chromosomal location, a miR cluster in Chr14q32 (Fig. 4c) showed important differences in connectivity between tumour (Fig. 4e) and control (Fig. 4d) data. A pathway analysis shows that EMT and TGF-beta pathways, crucial processes involved in breast cancer, are deregulated in our tumour samples. Finally, we assessed the presence of relevant miRs in our inferred networks with validated or previously predicted miR-mRNA edges. Given the above mentioned results and previous evidence we propose a series of hypotheses suggesting miRs influence breast cancer transcriptional regulation.

Despite the fact that networks inferred from control and tumour data were constructed in the same manner with data from the same 86 patients, there are noticeable differences between them; these differences seem to reflect transcriptional deregulation in the cancerous samples. For example, the larger number of miR and miR-miR edges for tumour data network might be considered as a gain of miR regulation. However, it seems not to be the case, as edges from networks inferred from control data tend to have higher MI values than the edges of the network inferred from tumour data (Fig. 1a,b). It is worth noticing that MI is a measure of statistical dependency, hence, higher MI values in controls may imply a stronger regulation between a few miRs over a large number of common mRNAs. Many of the of miR and miR-miR edges in the network inferred from tumour data could be affecting the specificity of an otherwise highly directed and organized miR regulation^9^, therefore favouring signalling that relates to phenotypic plasticity and heterogeneity proper of cancer cells^29^.

Network parameters suggest a miR involvement in network cohesion. miRs join different small components and incorporate mRNAs that are only connected to the network via miR-mRNA edges. Network miR removal results in thousands of mRNAs turning into single nodes and several components being created (Fig. 2e,f). Possessing less mRNA-mRNA edges and also lower MI values for said edges, could make the network inferred from tumour data more susceptible to disintegration, as miR absence resulted in the appearance of almost twice as many components compared to controls.

Affected specificity along with susceptibility to disintegration seem to account for the diversity found in the enrichment analysis results, as the number of enriched GO-terms for mRNAs from the network inferred from tumour data is much larger compared to the control (Supplementary Tables S3 and S4). These terms found from the tumour data network mRNAs are related to mechanisms known to participate in tumour promotion and survival, especially to tumour-extracellular matrix interactions^30^. In contrast, the mRNA enrichment results from the network inferred from control data show terms mainly associated to cellular maintenance and tissue homeostasis (Supplementary Table S2).

A deeper examination based on the highest degree nodes revealed that the miR-200 and the miR-199 highest degree miRs are determinant for the networks inferred from control and tumour data structure, respectively. Despite the fact that miR-200 miRs are present in tumour and control data networks, their associated networks show important differences in their structure. On the one hand, miR-200 first neighbours determine global architecture of controls network; on the other hand, tumour data network contains all miR-200 members but global structure is determined by miR-199 and their neighbours.

Regarding functionality, the aforementioned topological differences are reflected in the networks enrichment analysis results. In the miR-200 network inferred from control data (Fig. 3a) we observe that top GO-terms are related to cell adhesion, protein kinase activity and cellular junctions (Table 1). Meanwhile, regulation of extracellular space, molecule binding, immune system and developmental process related GO-terms are enriched in miR-200 network inferred from tumour data (Fig. 3b). Also, even with a smaller number of mRNAs, tumour data miR-200 network enriched for more terms than the control. Furthermore, miR-199 members and their first neighbours showed similar enrichment results to miR-200 family, regarding development and tumour-extracellular matrix regulation. This could suggest a cooperative relationship between miR-199, miR-200 and their neighbours in the tumour.

Our networks showed a group of conserved associations between the miR-200 networks inferred from tumour and control data, this association core is conformed by miR-200 miRs and known transcription factors such as: TWIST-1, TWIST-2, ZEB-1 and ZEB-2 (Fig. 3c). The presence of these common nodes and edges seems to be fundamental as miR-200 and aforementioned transcription factors have been associated with the acquisition of mesenchymal traits in epithelial cells by a process known as: Epithelial to Mesenchymal Transition (EMT)^19–21^ and the reverse process: Mesenchymal to Epithelial Transition (MET)^20^. EMT and MET are related to tissue development and maintenance, as in normal breast tissue, although, the traits obtained by cells that have undergone these programmes are important for tumour metastasis, invasiveness, immune suppression and the acquisition of cancer stem cell functionality^21,31^. EMT and MET programmes should not be depicted as binary switches between fully differentiated states^32^, but as the acquisition of traits in partially differentiated cells. The dynamic interconversion between epithelial and mesenchymal states associated with Epithelial-Mesenchymal Plasticity (EMP) is required for successful cancer cell metastatic colonization^32–35^.

Interestingly, miR-200 miRs are highly overexpressed meanwhile the majority of neighbouring mRNAs and said transcription factors are underexpressed (Fig. 3c). This expression profile seems to suggest a canonical interaction between miR-200 and possible targets, although our networks are undirected and the direction of the edges can not be assured. The particular expression profile of this core, suggests a crucial role for these miRs and their associations into the regulation of breast cancer associated mechanisms.

In concordance with miR-200 overexpression in our samples, MET and the acquisition of epithelial traits are key features for tumour colonization, pluripotency and self-renewal gene expression^22,23,36^. Our data comes from invasive primary tumours exclusively, where miR-200 is overexpressed as natural breast tissue architecture is lost likely by the presence of a larger proportion of epithelial cells^37^, however, the appearance of miR and mRNAs that have been experimentally associated to malignancy in our networks (Table 2) suggests an important role for a mixed state promoting not only invasion and migration but also allowing the colonization of cells promoting epithelial features. Results altogether suggest a dual behaviour for miR-200 and related molecules into regulating EMP^32,38^. In particular miR-141-3p, as it contributes to the majority of connections between miR-200 and their associated transcription factors (Fig. 3c).

Along with miR-200, miR-199 seems to be involved in breast cancer malignancy promotion. miR-199 is the miR family that is most connected in our breast cancer network, the difference in connectivity suggest a crucial role for these miRs as regulators of cancer related cell processes. Several studies have reported the importance of miR-199a and miR-199b downregulation for the migration and invasion of breast cancer cells^39,40^. The increased connectivity along with the finding that miR-199b-5p is underexpressed in our breast cancer samples compared to the breast cancer tissue show a possible mechanism involving miR-199 repression over gene targets that mediate tumour promotion. Although, miR-199b-3p and miR-199a show no important differences in their expression (<1 Log Fold Change). Several genes are thought to be behind the tumour suppressor activity associated to miR-199a/b in breast cancer, such as HER2^41^, PAK4/MEK/ERK^39^, and SWI/SNF^42^. The study published by^40^ shows that miR-199a-5p can alter the expression of EMT related genes such as CDH1, ZEB1 and TWIST. In our networks we found that miR-199a/b possessed significant associations with ZEB1 and TWIST1. Evidence supporting a miR-199a/b central role in the regulation of processes related to malignancy in cancer is emerging^43–45^, and our results support these miR importance in breast cancer. The high number of associations found in networks suggest that most targets are largely unexplored and their study could contribute to the understanding of breast cancer biology.

Another relevant feature revealed by this analysis, is that the network inferred from tumour data contains a large number of miRs mapping to DLK1-DIO3 (delta-like 1 homolog-deiodinase, iodothyronine 3) locus in chromosome 14q32. The DLK1-DIO3 cluster is under imprinting regulation, this region contains the paternally expressed genes DLK1, RTL1, and DIO3 and the maternally expressed ncRNAs (lncRNA, miR clusters, snoRNAs and pseudogenes)^46^. Aside from being subject to parental imprinting and epigenetic regulation, this mega cluster region is associated with polycomb repressive complex 2^47^. DLK1-DIO3 region is conversed across mammals and its transcripts are involved in developmental processes capable of affecting growth and differentiation^48^ and processes that modulate degenerative diseases^49^, neurological and metabolic functions^46^. The function and regulation of the DLK1-DIO3 region have implications for human disorders and cancer^46,50,51^.

It is known that miRs present in the DLK1-DIO3 region are implicated in EMT suppression. The proposed mechanism involves the repression of the miR-200 family, ZEB-1/2 and TWIST-1 signalling network^52^. For this reason DLK1-DIO3 miRs have been mostly labelled as tumour suppressors. Although, it has also been reported that miRs belonging to this region are downregulated in mammary cell lines and human breast carcinoma^52^. The presence of the Dlk1-DIO3 miRs is especially important as they have been observed to be downregulated in epithelial tumours^53^, and at the earliest stages of reprogramming; so it has been assumed that such downregulation may improve reprogramming efficiency^54^. Accordingly, these miRs are mainly underexpressed in our dataset (Supplementary Fig. S4), suggesting again an important relationship between the expression signature of the miRs from the network inferred from tumour data and EMT/MET.

We found that most of the miR in the cluster are underexpressed in contrast to the control, which suggests a tumour suppressive activity for at least some of the miR in this region. Evidence in other types of cancer support the possible supressive activity of these underexpressed cluster in breast cancer^55^. DLK1-DIO3 miR silencing is associated with morphological, molecular and functional changes related to EMT by a mechanism comprising TWIST1, BMI1, ZEB1/2, and miR-200 family miR^52^. The miR in this cluster may have implications for breast cancer patient prognosis as it has been reported for lung cancer patients^56^ and the evidence suggests they have an active role in the regulation of EMT/MET associated genes^52^. In our pathway deregulation analysis we found several miR from the DLK1-DIO3 cluster that shared MI associations with the genes that participate in the annotated EMT related pathways (Supplementary Figs S11–S13), supporting their involvement in the regulation of this process.

Our results suggest that miR-200 and miR from the DLK1-DIO3 cluster are involved in EMP; having gene-level information we further extended our analysis by including pathway level instances. Pathifier^24^ was implemented since it provides a context-specific deregulation score based on the expression profile of the genes in the pathways of interest for individual samples. Based on our networks results, we found pathways directly related to EMT in three different pathway databases. Those results highlight a specific underlying process that emerged from our network approach.

The altered expression of miRs in our inferred networks has a complex effect over gene regulation. These effects seem to impact network topology highlighting the emerging pivotal role of EMP and related miRs in breast cancer^31,32^. As an instance, Supplementary Figs S11–S13 show a more profound description level, since in them the inferred associations are integrated with EMT pathway deregulation. The network analysis that resulted in the miR-200 core and DLK1-DIO3 cluster was complemented with the pathway level networks (Fig. 5), which showed the involvement of widely known EMP drivers and marker genes, such as TGF-beta 1 (TGFB1)^57,58^, TGFBR2^58^, VIM^59^, CDH1^20,60^ and CDH2^61^. The results show deregulated pathways and enriched biological processes related to both EMT and MET in our breast cancer samples, supporting the observation that cancer cells exist in a mixed state with coexisting epithelial and mesenchymal traits^32^. EMP is emerging as a decisive feature for metastatic outgrowth in breast cancer animal models^62^; required not only for acquiring mesenchymal traits that promote disemination but allowing their reversion to an epithelial state in the metastatic site.

We identified several oncomiRs related to cancer promotion or suppression, depending on their expression signature. A list of those miRs along with their expression is presented in Table 2. Although for some of them (such as: miR-145, miR-100, miR-379, and miR-493) their relationship with breast cancer has been widely studied, for most of them it has not been clearly established. The consistency in the expression profile and target association exhibit by the miRs in Table 2 and the aforementioned miR families results relevant as miR sequence-based target prediction databases may be biased and experimentally validated interaction databases are incomplete.

The presented networks are a reconstruction of the associations between miR and mRNA from expression data. Considering that the associations we are reporting are obtained by an algorithm from sequencing data, their match with experimental and prediction information (Supplementary Table S17) is especially important for supporting of our results. We have to consider that due to the database limitations a true/false positive rate or similar measures would not be appropriate to assess our inferred networks. Also, by comparing our results to other methods for miR-target linear and non-linear association prediction, we found an important number of edges that matched our interactions. The implementation^15^ of the prediction algorithms we tested does not require as much computational resources as ARACNe, and they are contained in a user-friendly graphic interface. However, aside from target prediction our pipeline also infers miR-miR and mRNA-mRNA coexpression and regulatory associations, allowing us to model a more complete transcriptional landscape for our breast cancer samples. In particular matches between the edges inferred through different methodologies regarding miR-199 and DLK1-DIO3 suggest an important role for these miR and their association in breast cancer, although their exact contribution remains be explored.

The proposed methodology integrates different levels of information for creating a robust model that describes the regulatory landscape of miRs in breast cancer. Even if this MI based network approach can not infer the interaction direction or assure that these are indeed miR-target interactions, we successfully reconstructed associations that are known to be critical for processes promoting malignancy. As our results show, aside from the evident relationship between the most connected and clustered miRs with development and EMT/MET regulation, the general behaviour of miRs in the network also suggest an inherent general mechanism responsible for the phenotype differences between the cancerous tissue and controls.

We constructed miR-gene regulatory networks with sequencing data from breast invasive carcinoma patients, using a network approach we found the highly studied involvement of miR-200 in the acquisition of epithelial and mesenchymal traits through EMP in breast cancer, and suggested the participation of other miRs such as the miR-199 family members and the DLK1-DIO3 cluster. Our results were supported by experimental data, showing that we were able to identify *bona fide* miR-mRNA associations. Further, the comparison of our results with other miR-mRNA prediction algorithms showed an important overlap, specially for less studied miR associations in breast cancer, such as miR-199 and DLK1-DIO3 cluster miRs. This MI-based network approach remarks how a data-driven analysis is useful to understand the role of miR regulation over cancer-related processes without prior miR-target or sequence information, thus providing valuable information regarding miR regulation that might be difficult or extremely expensive to obtain; becoming beneficial for future experiment design.

## Methods

**Figure 6:**
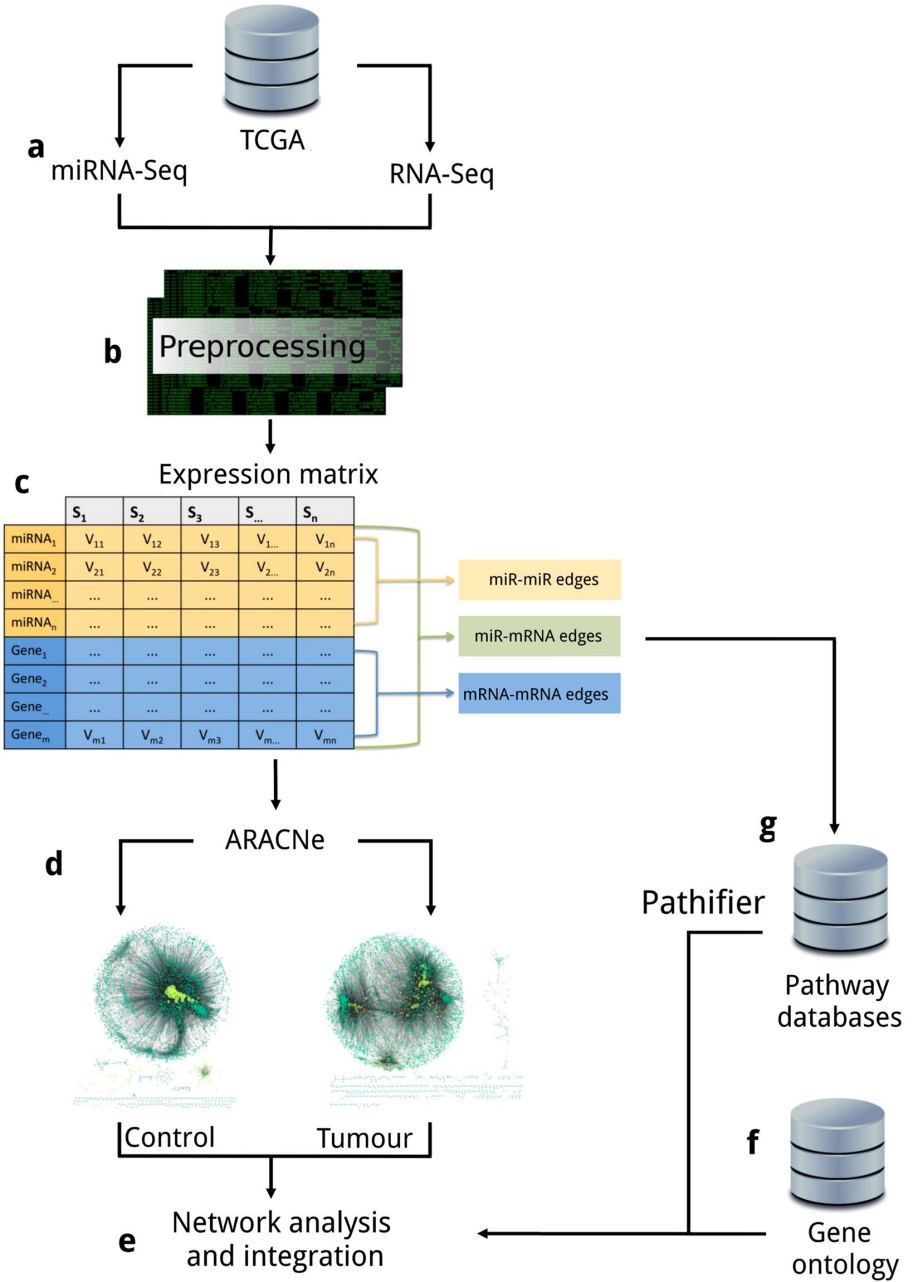
Pipeline description. (**a**) Paired RNAseq and miRseq expression data from 86 patients were obtained from TCGA. (**b**) Data preprocessing and normalization. (**c**) Normalized expression matrix with miR and mRNA (rows) expression values for each sample (columns). (**d**) Network construction by the ARACNE algorithm with the expression matrix as input. (**e**) Network analysis and result integration. (**f**) Gene ontology enrichment analysis from resulting network nodes (mRNAs). (**g**) Pathifier analysis from resulting network nodes (mRNAs) with first neighbour associations with miR-200 and DLK1-DIO3 miRs.

Level 3 RNA sequencing (RNAseq) (Illumina HiSeq. 2000 RNA Sequencing Version 2 analysis) and miR sequencing (miRNAseq) (Illumina HiSeq. 2000 miR Sequencing) from 86 primary breast invasive carcinoma patients was obtained from TCGA (Fig. 6a). In order to control the variability in the comparisons between control and tumour data we decided to only select patients with adjacent tissue controls matched to the tumour, with RNAseq and miRseq data for both samples.

### miR data pre-processing

We calculated mature miR counts according to the methodology recommended in BCGSC miR Profiling Pipeline Documentation^63^ according to miRBase 21^64,65^ with TCGA miR isoform information. Once mature counts were calculated, miRs with less than 5 counts in the 25% of the samples were filtered from the analysis^66^ and the remaining sets were normalized by the “Trimmed mean of M normalization” (TMM)^67^ contained in EdgeR (v.3.12.0) R package^68^ (Fig. 6b).

### RNAseq data pre-processing

Version 2 analysis of the TCGA RNAseq Illumina platform consists in “RNA-Seq by Expectation-Maximization” (RSEM) normalization^69^. Upper quartile normalized RSEM gene count estimates (transcripts per million (TPM) were used for the analysis, and filtered for RNAs with less than 10 TPM per sample (in average). From RNAseq and miRseq normalized datasets, we constructed expression matrices that were joined together; maintaining the matched TCGA patient code for its further analysis (Fig. 6c). This joint expression matrix was used as input for network construction.

### Network construction

For a given phenotype, genes that exhibit a similar transcriptional response are likely to be part of a common biological process. Reverse engineering gene networks represent transcripts regulatory associations as a graph, called a Gene Regulatory Network^70^. ARACNe (Algorithm for the Reconstruction of Accurate Cellular Networks)^25,71^ is a widely used information-theoretic algorithm that accounts for mRNAs as nodes, and associations (edges) as the statistical dependency between gene expression profiles, correlating pairs of mRNAs by means of their MI measure. MI values are directly related to the degree of statistical dependency between pairs of mRNAs, thus the direction of the associations can not be inferred only from this methodology.

It is worth mentioning that differences in miR and mRNA dynamic range do not preclude the analysis, as ARACNe uses a Gaussian Kernel estimator (GKE). The GKE decreases the influence of arbitrary transformations on the input data and removes the need for position-dependent kernel widths used in uniformly distributed data^25^. There may, however, be a kernel width effect on MI estimation: when the number of samples approaches infinite, the GKE is asymptotically unbiased, while for a finite sample number there is not a universal kernel width. To overcome this, ARACNe was designed to rely on MI ranks instead of the MI estimate accuracy^25^, and thus the associations inferred in our networks should not be affected by differences in miR and mRNA transcript abundance.

A parallelized implementation of ARACNe2 algorithm^72^ and MINET (v.3.28.0) R package^73^ were used to construct the network by computing the MI between all mRNAs and miRs in the dataset (Fig. 6d). A critical step in network construction is the selection of variables (mRNA, miR) that are more representative of the phenomenon (feature selection). For MI-based networks there is a relationship between MI value, number of samples and network statistical significance^74^.

We found that the distribution of MI for miR-miR and miR-mRNA edges tended to be smaller than mRNA-mRNA edges, and decided to prioritize miR-miR and miR-mRNA edges over mRNA-mRNA edges by applying a slightly less stringent threshold. From the normalized transcript abundance matrices, MI-based undirected networks were generated, and interactions were pruned using an MI threshold at the 99.987%ile of mRNA-mRNA edges (Bonferroni corrected *p*-value for control mRNA-mRNA edges = 7.12 × 10^−26^; for tumour mRNA-mRNA edges = 1.57 × 10^−19^), and the 99.741%ile of miR-miR and miR-mRNA edges (Bonferroni corrected *p*-value for control miR-miR and miR-mRNA edges = 1.12 × 10^−11^; for tumour miR-miR and miR-mRNA edges = 8.22 × 10^−3^). These *p*-value thresholds were used with a data processing inequality (DPI) tolerance threshold value of 10% as a reasonable trade-off between false negatives and false positives, conserving triplets that have similarly strong edges^74^.

Reported *p*-value thresholds were chosen such that all network edges were statistically significant and the resultant number of mRNA transcripts and miR edges were similar for tumour and control tissue inferred networks (Supplementary Table S1). These *p*-values correspond to 25,334 miR-miR and miR-mRNA edges and 14,892 mRNA-mRNA edges (before DPI pruning). We constructed networks with different *p*-value thresholds to test the network susceptibility of the edge inference this threshold value (Supplementary methods), the networks with different cut-offs show similar attributes and behaviours to the network reported within the results (Supplementary Tables S21–S28).

Network edges represent statistical dependence between pairs of expression profiles, this property is particularly useful to reconstruct canonical and non-canonical co-expression relationships between miR and mRNA. Without having to assume linear behaviour for their associations (e.g. Pearson correlation between miR and targets) or applying *a priori* biological criteria, especially since most interactions and their nature are widely unknown. To follow this methodology acquires crucial relevance in the case of miR associations because their overall functionality remains to be discovered.

### Network analysis

To study the network topological properties and visualize resulting networks (Fig. 6e) we used Cytoscape^75^. The analysis focused on the degree centrality measure, stressing on the most connected miR and their first neighbours (nodes directly connected). Visualizations were obtained with the spring-embedded algorithm. Hiveplot visualization was performed according to^76^ in order to provide a comparable network layout. The alluvial diagram was created through the RAWGraphs web-tool (http://rawgraphs.io/).

### Differential expression analysis

Differential expression analysis of RNASeq gene results and mature miRSeq raw counts were performed by using DESeq. 2 (v.1.10.1) R package^77^. Differentially expressed genes were used to identify important nodes with a possible relevant biological role, a miR or gene was considered as differentially expressed if their expression changed at least twofold compared to controls and had a Benjamini-Hochberg adjusted *p*-value < 0.01. The network construction pipeline can be found at a GitHub repository hosting service (https://github.com/CSB-IG/miRseq_rnw). Analyses were mainly conducted in R programming language (v.3.2.0).

### Functional analysis

#### Enrichment analysis: Gene Ontology

To study the biological significance of the networks structure and properties, a functional enrichment analysis was performed. We used the BiNGO Cytoscape plug-in (Fig. 6f) to analyse the genes corresponding to the mRNA nodes in the networks; this tool gives relevant information regarding ontologies of biological process, molecular function and cellular component categories. Biological process involves pathways and cellular processes, molecular function refers to gene products activities, and cellular component to the location where gene products are active. As an over representation analysis, the statistical significance of the association is calculated by a hypergeometric test. This method uses the the input gene and the database collections information from both to calculate the probability of certain genes to pertain to a specific process. We decided to centre on the most significant GO terms (FDR < 0.01) that are constituted by less than 1,000 genes.

#### Functional Class Scoring: Pathifier

Pathifier^24^ is a Functional Class Scoring algorithm; which means that, in contrast to over-representation analyses, it uses all the available measurements of experimental high-throughput biological data to evaluate their enrichment scores^78^. The Pathifier algorithm performing a principal component analysis, evaluates a certain pathway expression data into a coordinate system creating a cloud of points. Afterwards, using the Hastie and Stueltzle’s algorithm (Hastie and Stuetzle, 1989) the points are used to calculate a principal curve, using as initial point of the centroid the control samples. Finally, the samples are projected to the closest point to the principal curve. Pathifier uses a metric named as Pathway Deregulation Score (PDS), that corresponds to the relative distance from the sample projection to the initial point centroid^24^.

We used Pathifier algorithm restring the pathways to the ones that covered our criteria (Fig. 6g). We made a selection of the pathways in WikiPathways, Reactome and KEGG that contained at least a gene present in the miR-200 first neighbour network inferred from tumour data; and the pathways from the same databases that contained at least a gene present in the DLK1-DIO3 miR cluster first neighbour network inferred from tumour data. From the selected pathways we filtered pathways with less than 4 genes and those with more genes than our number of samples.

We used the CytoKEGG, Reactome FI^79^ and WikiPathways^80^ Cytoscape plugins to create the network visualization of the pathways of interest. We merged the pathways with our inferred networks to find relevant miR involvement in the pathways.

#### Validated and Predicted Interactions: TargetScan & miRTarBase

For miR functional enrichment, we resort to a literature search for miR biological relationships, since there are few integrative tools available to analyse them. In order to study miR that might have a stronger biological relevance for the phenotype we selected miR from the most connected miR family in the networks and their first neighbours to be enriched. We used the available information from the experimentally validated interactions in miRTarBase^27^ and the miR-target prediction associations from TargetScan^28^, to explore for the presence of common interactions between the databases and our inferred networks using Cytoscape^75^.

#### miR-mRNA inference comparison

We used miRTarVis:^15^ MI^81^, Maximal Information-based Nonparametric Exploration (MINE)^82^, Pearson correlation, and GenMiR +  + ^18^ miR-mRNA prediction implementations to calculate the top miR-mRNA associations from our data, and compared them to our tumour data network. We used DESeq. 2^77^ (v.1.10.1) for variance stabilization of the miR and mRNA raw expression data. Processed data was loaded in the miRTarVis^15^ interactive interface and prediction algorithms were fixed to a top number of interactions, for the network inferred from tumour data we used the top 40,316 associations for each method, 4,186 for DLK1-DIO3 miR and 1,535 for miR-199. The number of top interactions was fixed to match the number of miR-mRNA associations obtained for miR-199 and DLK1-DIO3 miR by our network inference methodology, for the tumour data network it was chosen to match the initial number of interactions (before DPI prunning).

## Electronic supplementary material

Supplementary Information

Table S3

Table S4

Table S7

Table S8

Table S10

Table S11

Table S12

Table S13

Table S14

Table S15

Table S16

Table S19

Table S20
