# Supplementary material for: Network analysis of EMT and MET micro-RNA regulation in breast cancer
Source: Sci Rep. 2017 Oct 19;7:13534. doi: 10.1038/s41598-017-13903-1 (PMC5648819; doi:10.1038/s41598-017-13903-1)
Supplement: Supplementary file 1 — Supplementary Information [file 41598_2017_13903_MOESM1_ESM.pdf]

Network Analysis of EMT and MET micro-RNA Regulation in Breast Cancer

Diana Drago-García[1], **Jesús Espinal-Enríquez**[1,2], **and Enrique Hernández-Lemus**[1,2,*]

[1]Computational Genomics Division, National Institute of Genomic Medicine (INMEGEN), 14610, México
[2]Centro de Ciencias de la Complejidad, Universidad Nacional Autónoma de México (UNAM), 04510 México
[*]ehernandez@inmegen.gob.mx

Supplementary Information

Supplementary Information Index

Supplementary Tables

Table	S 1	Network attributes	Supplementary information	page 3
Table	S 2	Top enrichment results for tumour and control networks	Supplementary information	page 7
Table	S 3	Control data GO enrichment results	xml format	Table_S3.xml
Table	S 4	Tumour data GO enrichment results	xml format	Table_S4.xml
Table	S 5	Top ten high-degree nodes in tumour and control networks	Supplementary information	page 7
Table	S 6	miR-200 family members degree in first neighbour networks	Supplementary information	page 8
Table	S 7	Control data miR-200 subnetwork GO enrichment results	xml format	Table_S7.xml
Table	S 8	Tumour data miR-200 subnetwork GO enrichment results	xml format	Table_S8.xml
Table	S 9	miR-199 family members degree in first neighbour networks	Supplementary information	page 8
Table	S 10	Tumour data miR-199 subnetwork GO enrichment results	xml format	Table_S10.xml
Table	S 11	Pathway deregulation score matrix: DLK1-DIO3 genes analysis (Reactome)	xml format	Table_S11.xml
Table	S 12	Pathway deregulation score matrix: DLK1-DIO3 genes analysis (WikiPathways)	xml format	Table_S12.xml
Table	S 13	Pathway deregulation score matrix: DLK1-DIO3 genes analysis (KEGG)	xml format	Table_S13.xml
Table	S 14	Pathway deregulation score matrix: miR-200 genes analysis (Reactome)	xml format	Table_S14.xml
Table	S 15	Pathway deregulation score matrix: miR-200 genes analysis (Wikipathways)	xml format	Table_S15.xml
Table	S 16	Pathway deregulation score matrix: miR-200 genes analysis (KEGG)	xml format	Table_S16.xml
Table	S 17	Validated and predicted miR-mRNA associations (Full network results)	Supplementary information	page 19
Table	S 18	Validated and predicted miR-mRNA associations (miR-200 subnetwork results)	Supplementary information	page 19
Table	S 19	miRTarVis matching edges with inferred network results (DLK1-DIO3 associations)	xml format	Table_S19.xml
Table	S 20	miRTarVis matching edges with inferred network results (miR-199 associations)	xml format	Table_S20.xml
Table	S 21	Variation on networks p-value threshold: network attributes	Supplementary information	page 21
Table	S 22		Supplementary information	page 21
Table	S 23		Supplementary information	page 22
Table	S 24		Supplementary information	page 22
Table	S 25	Variation on networks p-value threshold: top ten high-degree nodes	Supplementary information	page 22
Table	S 26		Supplementary information	page 23
Table	S 27		Supplementary information	page 23
Table	S 28		Supplementary information	page 23

Supplementary Figures

Figure S 1	Tumour and control networks MI distribution Q-Q plot	Supplementary information	page 3
Figure S 2	Whole network visualization: Control data	Supplementary information	page 5
Figure S 3	Whole network visualization: Tumour data	Supplementary information	page 6
Figure S 4	DLK1-DIO3 miRs network visualization: Tumour data	Supplementary information	page 9
Figure S 5	Pathway deregulation score heatmap: DLK1-DIO3 genes analysis (Reactome)	Supplementary information	page 10
Figure S 6	Pathway deregulation score heatmap: DLK1-DIO3 genes analysis (WikiPathways)	Supplementary information	page 11
Figure S 7	Pathway deregulation score heatmap: DLK1-DIO3 genes analysis (KEGG)	Supplementary information	page 12
Figure S 8	Pathway deregulation score heatmap: miR-200 genes analysis (Reactome)	Supplementary information	page 13
Figure S 9	Pathway deregulation score heatmap: miR-200 genes analysis (Wikipathways)	Supplementary information	page 14
Figure S 10	Pathway deregulation score heatmap: miR-200 genes analysis (KEGG)	Supplementary information	page 15
Figure S 11	Network visualization: TGF-beta receptor siganling in EMT (Reactome)	Supplementary information	page 16
Figure S 12	Network visualization: TGF-beta signaling (KEGG)	Supplementary information	page 17
Figure S 13	Network visualization: TGF-beta receptor signaling in thyroid cells for EMT (WikiPathways)	Supplementary information	page 18
Figure S 14	Network visualization: TargetScan and miRTarBase matching edges with inferred networks	Supplementary information	page 20

Supplementary Methods

Network construction: variation on network's p-value threshold strategy	Supplementary information	page 21

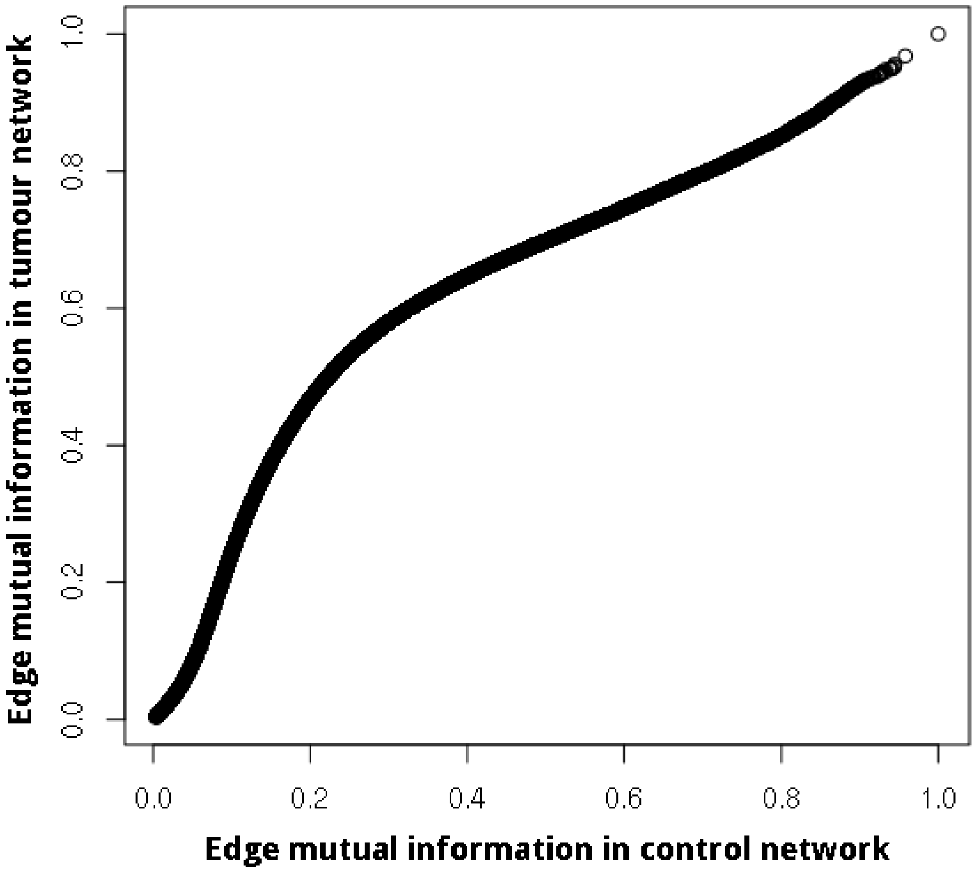

Figure S1. Q-Q plot of the whole set of MI interactions for networks inferred from tumour and control data.

Table S1. Attributes of the Networks inferred from control and tumour data (Lc: Largest Component).

Attribute	Whole network		Largest component		Lc mRNA-mRNA subnetwork	
	Control	Tumour	Control	Tumour	Control	Tumour
Total nodes	4,575	4,602	4,229	4,200	4,096	3,714
miR	241	514	133	486	0	0
mRNA	4,334	4,088	4,096	3,714	4,096	3,714
Total edges	33,879	29,186	33,388	28,913	14,486	11,010
miR-miR	482	1,775	240	1,769	0	0
mRNA-miR	18,760	16,173	18,662	16,134	0	0
mRNA-mRNA	14,637	11,238	14,486	11,010	14,486	11,010
Components	102	165	1	1	1,856	2,590
Single nodes	0	0	0	0	1,814	2,524

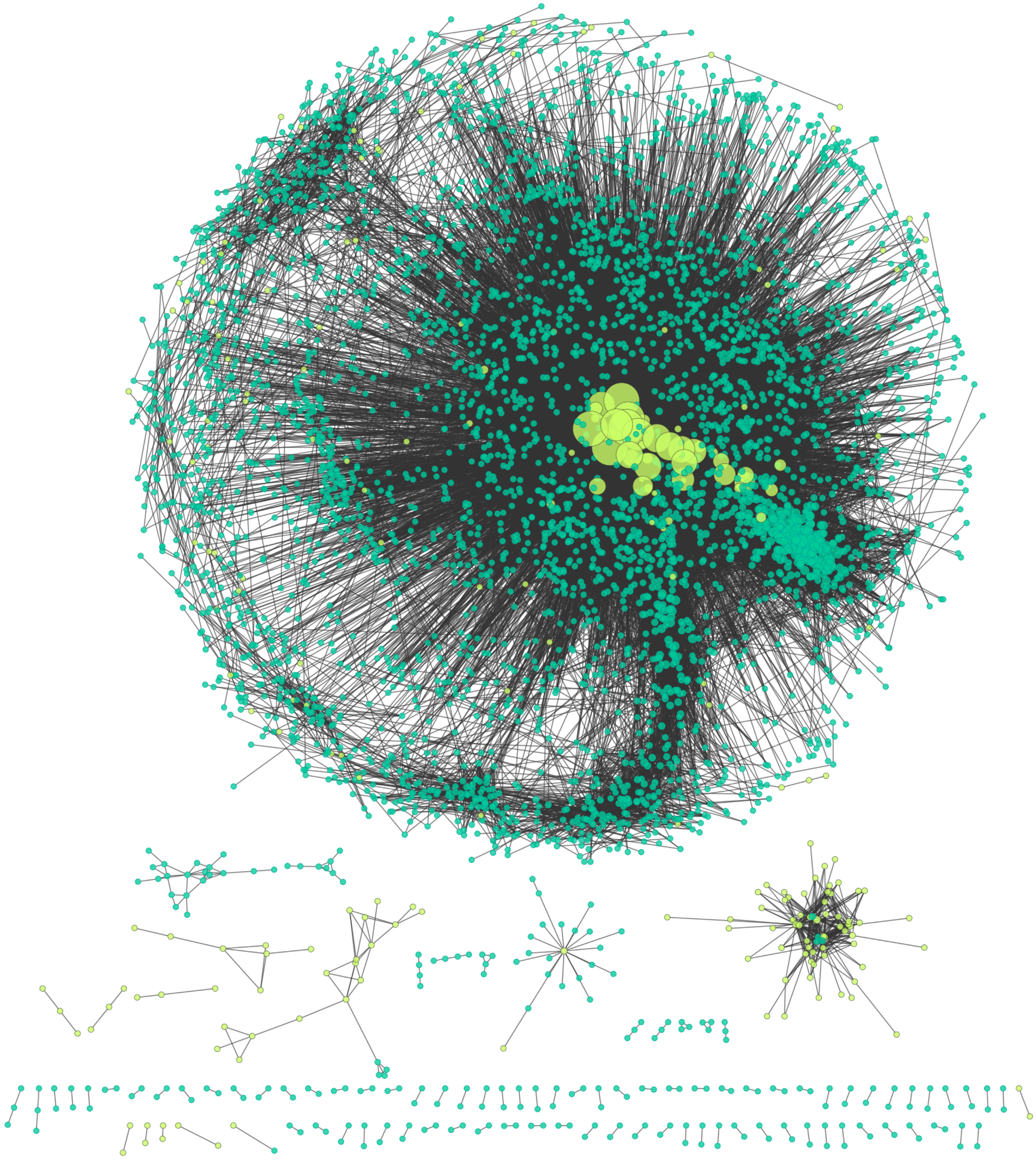

Figure S2. Spring embedded visualization of the whole network inferred from control data. In this figure miRs are represented as green nodes, and mRNAs as turquoise nodes.

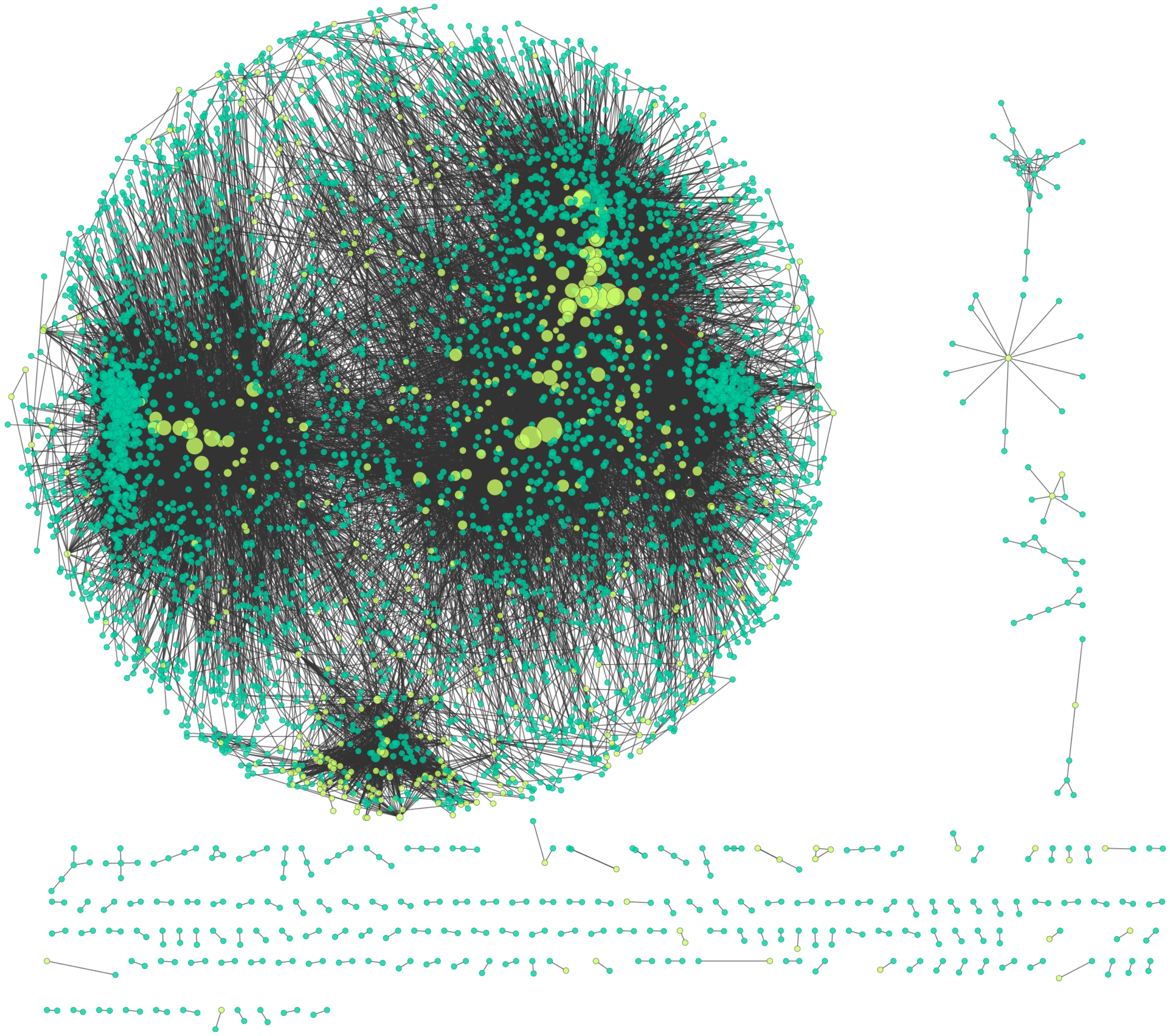

Figure S3. Spring embedded visualization of the whole network inferred from tumour data. In this figure miRs are represented as green nodes, and mRNAs as turquoise nodes.

Table S2. Enrichment analysis top results for tumours and controls networks. The left column shows the Gene Ontology categories and their respective enriched terms, meanwhile the right column contains the related statistical significance (FDR: False Discovery Rate, Benjamini-Hochberg corrected p-value < 0.01).

**Controls**		**Tumours**	
	FDR		FDR
**Biological process**		**Biological process**	
Translational elongation	5.4716×10^{-19}	Immune system process	1.3985×10^{-56}
Phosphorylation	7.5397×10^{-6}	Immune response	1.9804×10^{-44}
rRNA transcription	1.0708×10^{-4}	Regulation of immune system process	4.0949×10^{-44}
Oxidation reduction	1.4061×10^{-4}	Cell adhesion	2.0828×10^{-37}
Protein amino acid phosphorylation	1.4996×10^{-4}	Biological adhesion	2.4522×10^{-37}
**Molecular function**		**Molecular function**	
Transferase activity, transferring phosphorus-containing groups	6.2019×10^{-11}	Cytokine binding	6.3558×10^{-16}
Kinase activity	3.2346×10^{-10}	Carbohydrate binding structural constituent	1.8109×10^{-13}
Phosphotransferase activity, alcohol group as acceptor	6.1591×10^{-8}	Polysaccharide binding	2.2194×10^{-13}
Enzyme binding	2.3155×10^{-7}	Pattern binding	2.2194×10^{-13}
GTPase regulator activity	9.8384×10^{-7}	Glycosaminoglycan binding	2.8339×10^{-13}
**Cellular component**		**Cellular component**	
Cytosolic ribosome	4.2300×10^{-20}	Extracellular region part	7.3601×10^{-50}
Cell-cell junction	1.1648×10^{-11}	Extracellular matrix	3.3204×10^{-36}
Cytosolic part	6.2019×10^{-11}	Proteinaceous extracellular matrix	6.5129×10^{-34}
Ribosomal subunit	1.2319×10^{-9}	Extracellular space	5.3344×10^{-31}
Cytosolic small ribosomal subunit	1.5364×10^{-9}	Extracellular matrix part	1.7167×10^{-16}

Table S5. Top ten high degree nodes in networks inferred from control and tumour data.

**Control**		**Tumour**	
Node	Degree	Node	Degree
hsa-mir-200b-3p	1,445	hsa-let-7c-5p	509
hsa-mir-200a-3p	1,437	hsa-mir-199a-5p	500
hsa-mir-141-5p	1,392	hsa-mir-199b-5p	498
hsa-mir-141-3p	1,363	hsa-mir-337-3p	446
hsa-mir-200a-5p	1,135	hsa-mir-99a-5p	419
hsa-mir-200c-3p	1,106	hsa-mir-134-5p	342
hsa-mir-193b-5p	1,086	hsa-mir-199a-3p	319
hsa-mir-652-3p	770	hsa-mir-199b-3p	318
hsa-mir-22-3p	769	hsa-mir-382-5p	311
hsa-mir-378a-5p	631	hsa-mir-223-3p	285

Table S6. mir-200 family miRs degree in the first neighbour networks inferred from control and tumour data.

	**Control**	**Tumour**
miR	Degree	
hsa-mir-200a-3p	1,437	16
hsa-mir-200a-5p	1,135	2
hsa-mir-200b-3p	1,445	18
hsa-mir-200b-5p	291	17
hsa-mir-200c-3p	1,106	53
hsa-mir-200C-5p	641	7
hsa-mir-141-5p	1,392	5
hsa-mir-141-3p	1,363	181
hsa-mir-429	367	4

Table S9. mir-199 family miRs degree the in first neighbour networks inferred from control and tumour data.

	**Control**	**Tumour**
miR	Degree	
hsa-mir-199a-5p		500
hsa-mir-199a-3p	1	319
hsa-mir-199b-5p		498
hsa-mir-199b-3p	1	318

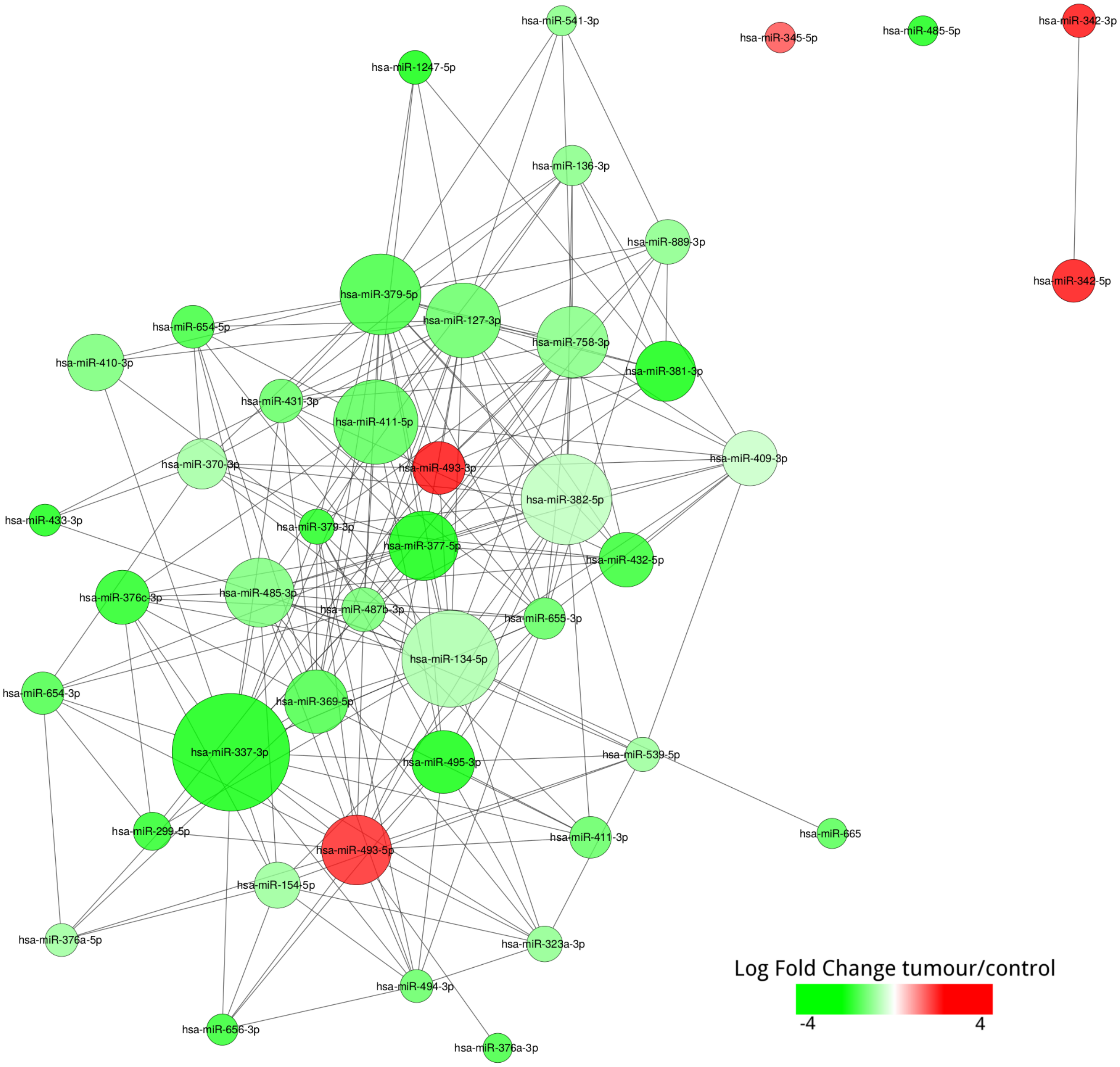

Figure S4. Differential expression for miRs from DLK1-DIO3 region (tumour data network). It should be noted that the majority of miRs are underexpressed.

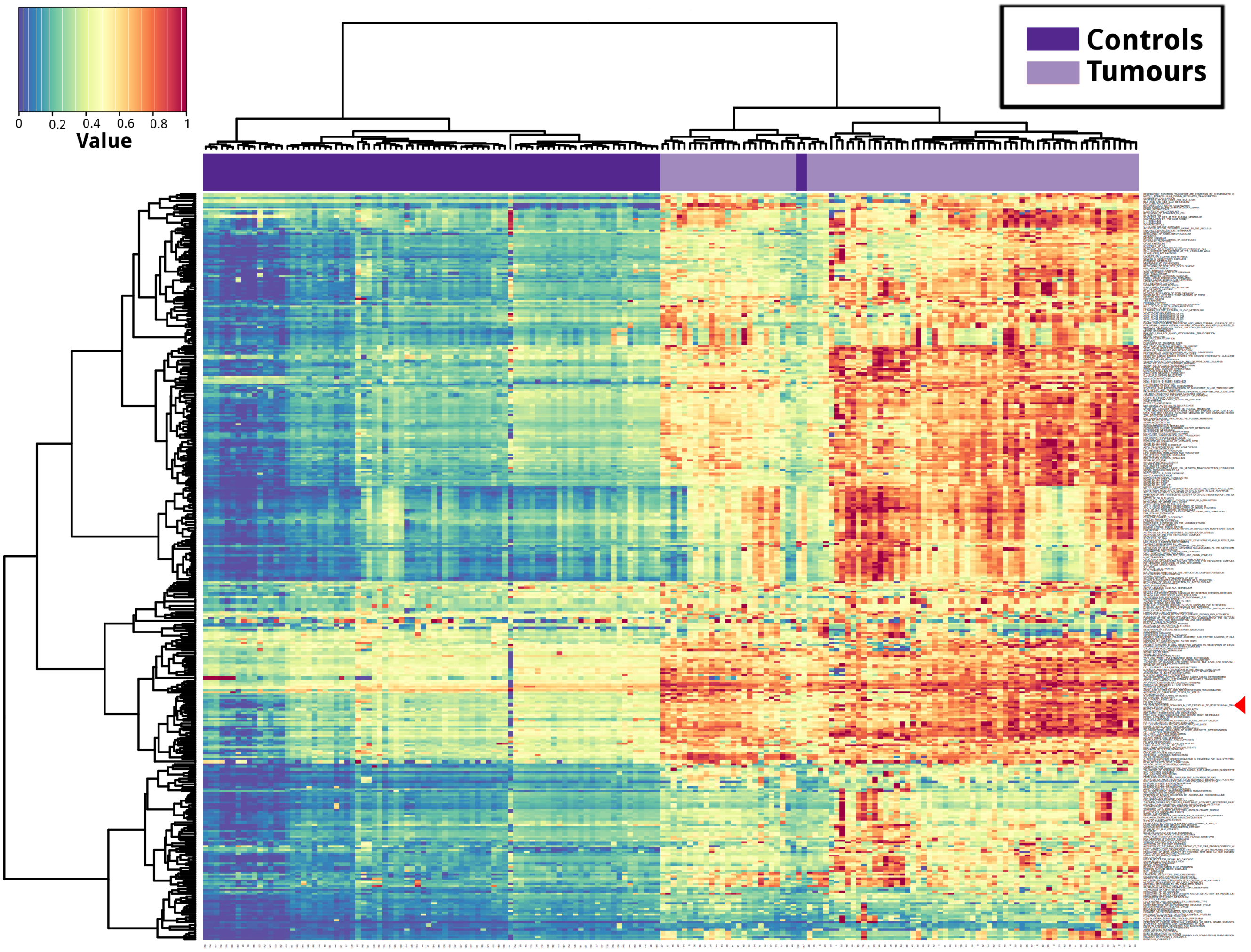

Figure S5. Pathway Deregulation Score heatmap for the Reactome pathways that contained a least a gene involved in first neighbours associations with the miRs in the DLK1-DIO3 cluster in our network inferred from tumours data. In this figure samples are represented as columns and the pathways included in the analysis as rows. The figure contains the Pathway Deregulation Scores (PDS) obtained for 393 Reactome pathways for 172 samples (86 tumour samples and 86 matching control samples) coloured according to the color code at the top left of the figure. Which means that blue-green pathways are slightly deregulated, and orange ones are strongly deregulated. Blue and Red bars at the top of the heatmap represent control and tumour samples, respectively. Euclidean distances and the Ward hierarchical clustering method were used for the dendogram. The red arrow points the pathway: TGF-beta receptor signalling in EMT (epithelial to mesenchymal transition).

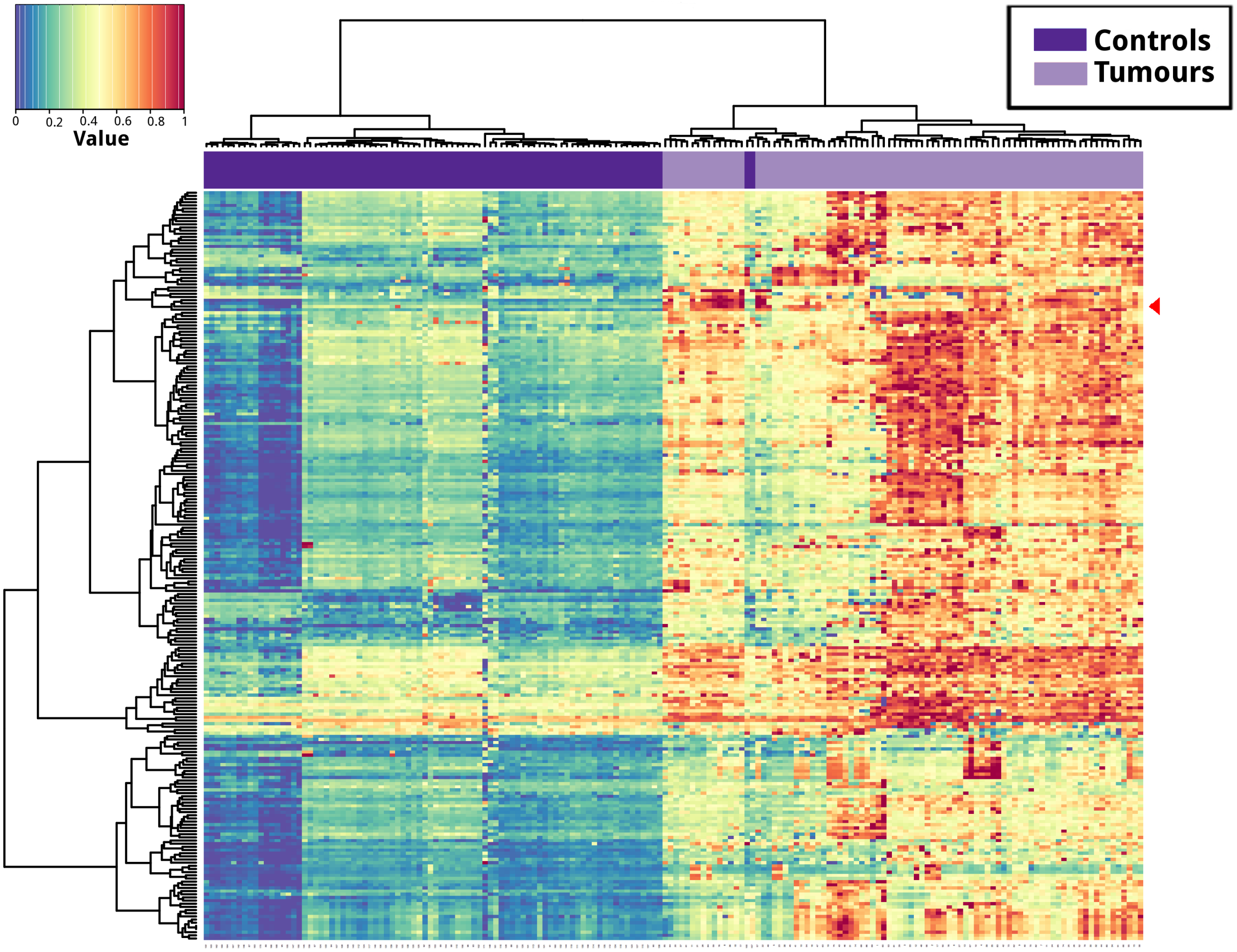

Figure S6. Pathway Deregulation Score heatmap for the WikiPathways pathways that contained a least a gene involved in first neighbours associations with the miRNAs in the DLK1-DIO3 cluster in our network inferred from tumours data. In this figure samples are represented as columns and the pathways included in the analysis as rows. The figure contains the Pathway Deregulation Scores (PDS) obtained for 237 WikiPathways pathways for 172 samples (86 tumour samples and 86 matching control samples) coloured according to the color code at the top left of the figure. Which means that blue-green pathways are slightly deregulated, and orange ones are strongly deregulated. Blue and Red bars at the top of the heatmap represent control and tumour samples, respectively. Euclidean distances and the Ward hierarchical clustering method were used for the dendogram. The red arrow points the pathway: TGF-B Signaling in Thyroid Cells for Epithelial-Mesenchymal Transition.

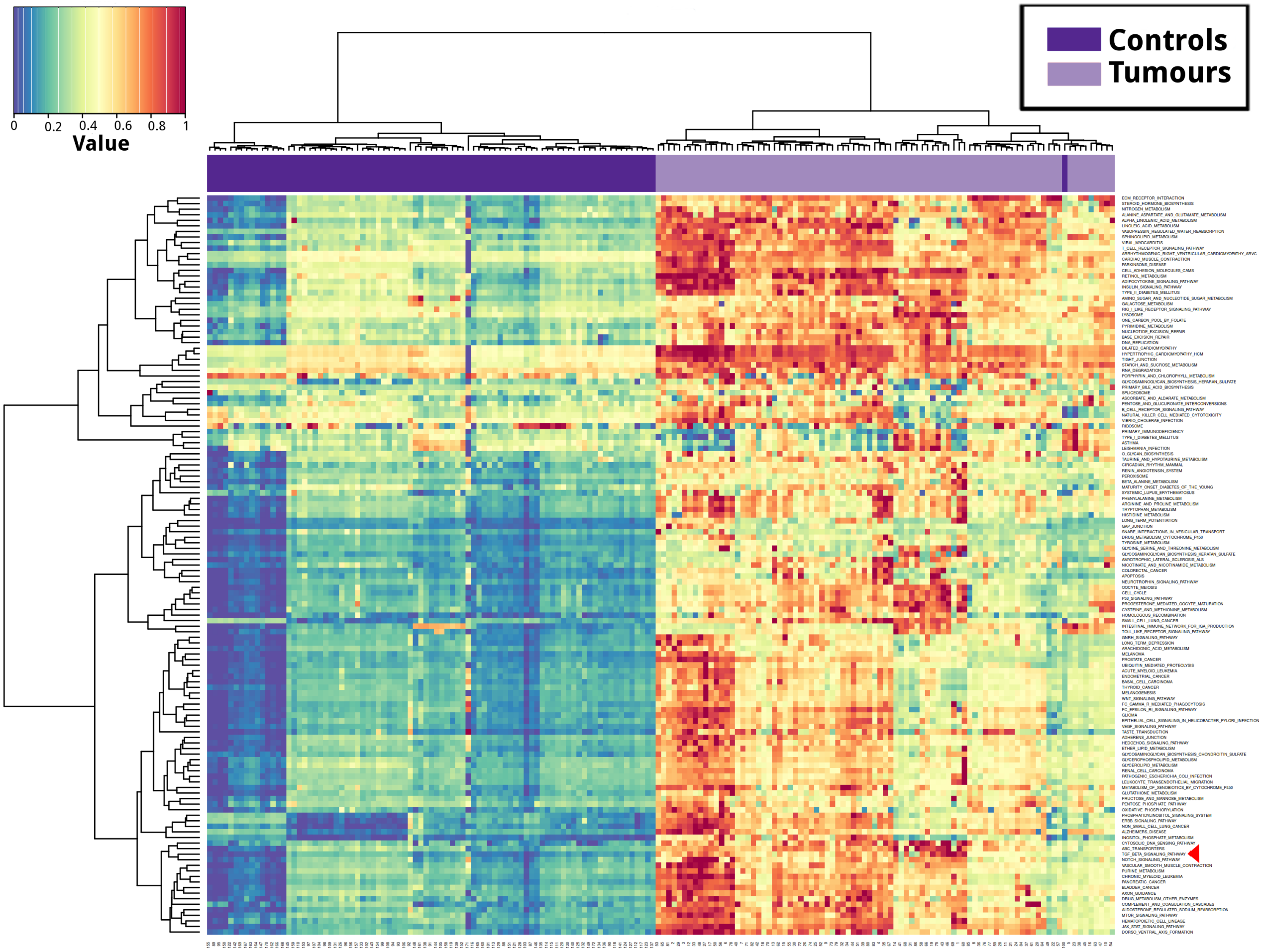

Figure S7. Pathway Deregulation Score heatmap for the KEGG pathways that contained a least a gene involved in first neighbours associations with the miRNAs in the DLK1-DIO3 cluster in our network inferred from tumours data. In this figure samples are represented as columns and the pathways included in the analysis as rows. The figure contains the Pathway Deregulation Scores (PDS) obtained for 133 KEGG pathways for 172 samples (86 tumour samples and 86 matching control samples) coloured according to the color code at the top left of the figure. Which means that blue-green pathways are slightly deregulated, and orange ones are strongly deregulated. Blue and Red bars at the top of the heatmap represent control and tumour samples, respectively. Euclidean distances and the Ward hierarchical clustering method were used for the dendogram. The red arrow points the pathway: TGF-beta signaling.

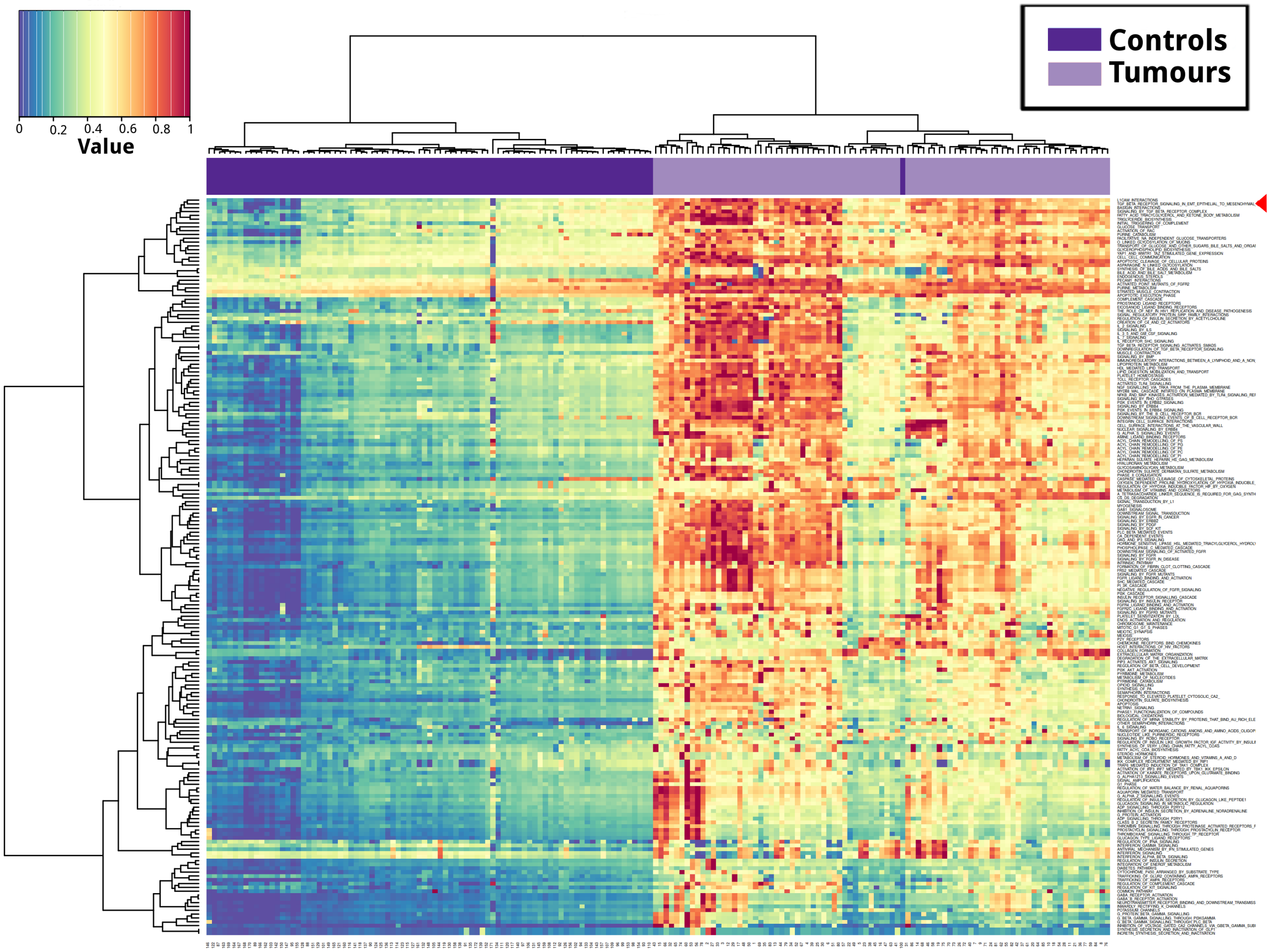

Figure S8. Pathway Deregulation Score heatmap for the Reactome pathways that contained a least a gene involved in first neighbours associations with the miR-200 family in our network inferred from tumours data. In this figure samples are represented as columns and the pathways included in the analysis as rows. The figure contains the Pathway Deregulation Scores (PDS) obtained for 193 Reactome pathways for 172 samples (86 tumour samples and 86 matching control samples) coloured according to the color code at the top left of the figure. Which means that blue-green pathways are slightly deregulated, and orange ones are strongly deregulated. Blue and Red bars at the top of the heatmap represent control and tumour samples, respectively. Euclidean distances and the Ward hierarchical clustering method were used for the dendogram. The red arrow points the pathway: TGF-beta receptor signaling in EMT (epithelial to mesenchymal transition).

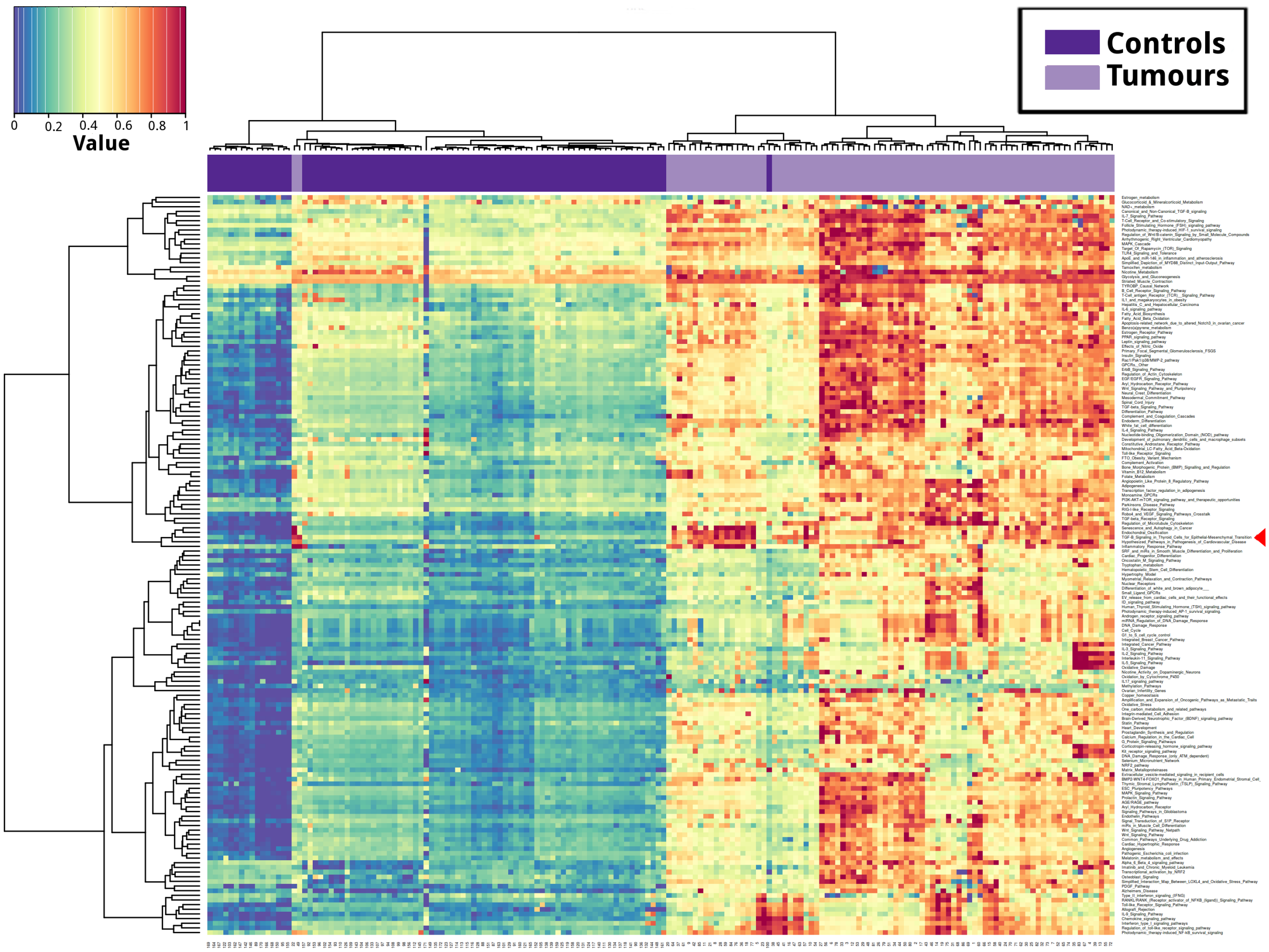

Figure S9. Pathway Deregulation Score heatmap for the WikiPathways pathways that contained a least a gene involved in first neighbours associations with the miR-200 family in our network inferred from tumours data. In this figure samples are represented as columns and the pathways included in the analysis as rows. The figure contains the Pathway Deregulation Scores (PDS) obtained for 159 WikiPathways pathways for 172 samples (86 tumour samples and 86 matching control samples) coloured according to the color code at the top left of the figure. Which means that blue-green pathways are slightly deregulated, and orange ones are strongly deregulated. Blue and Red bars at the top of the heatmap represent control and tumour samples, respectively. Euclidean distances and the Ward hierarchical clustering method were used for the dendogram. The red arrow points the pathway: TGF-B Signaling in Thyroid Cells for Epithelial-Mesenchymal Transition.

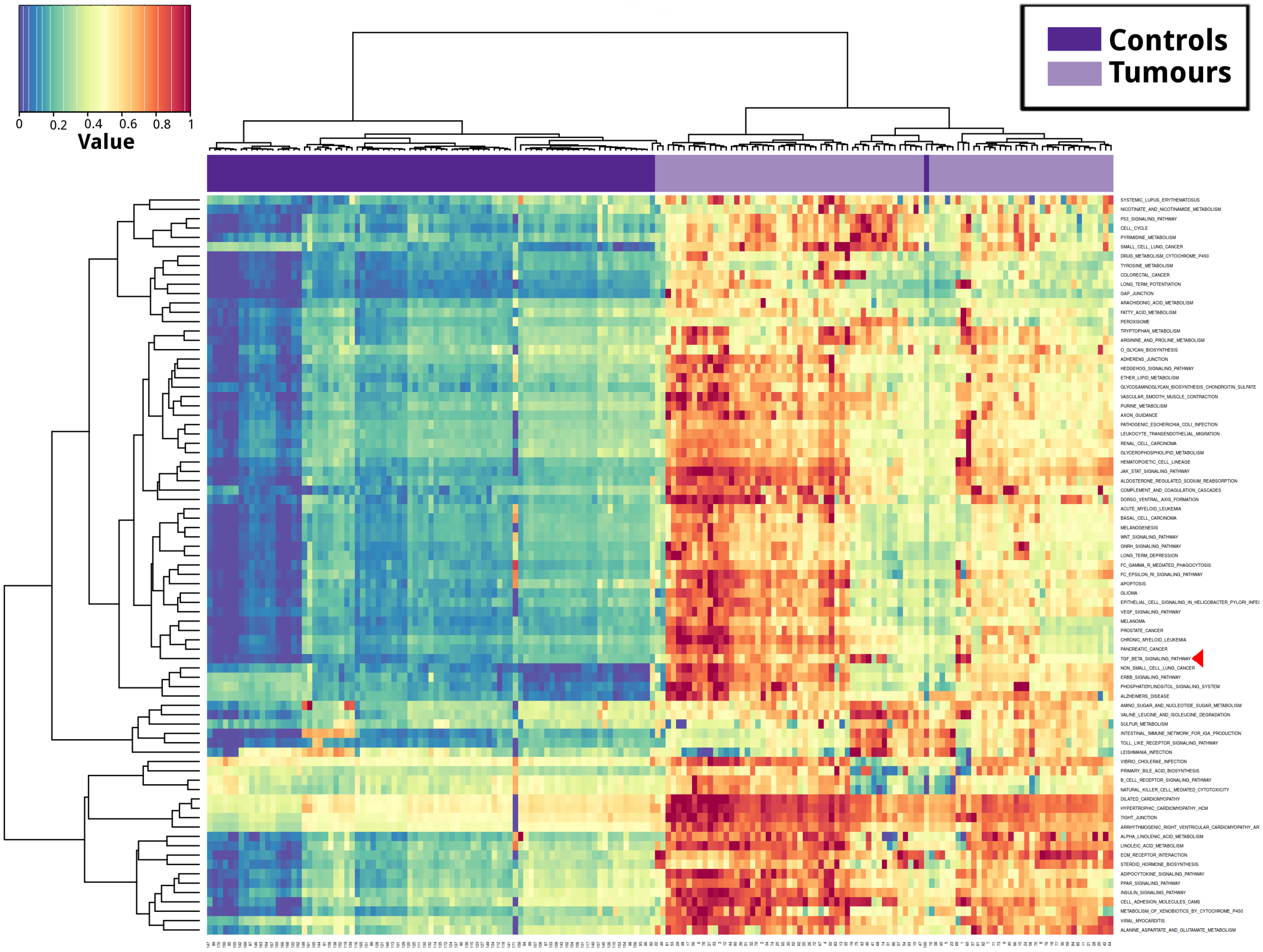

Figure S10. Pathway Deregulation Score heatmap for the KEGG pathways that contained a least a gene involved in first neighbours associations with the miR-200 family in our network inferred from tumours data. In this figure samples are represented as columns and the pathways included in the analysis as rows. The figure contains the Pathway Deregulation Scores (PDS) obtained for 79 KEGG pathways for 172 samples (86 tumour samples and 86 matching control samples) coloured according to the color code at the top left of the figure. Which means that blue-green pathways are slightly deregulated, and orange ones are strongly deregulated. Blue and Red bars at the top of the heatmap represent control and tumour samples, respectively. Euclidean distances and the Ward hierarchical clustering method were used for the dendogram. The red arrow points the pathway: TGF-beta signaling.

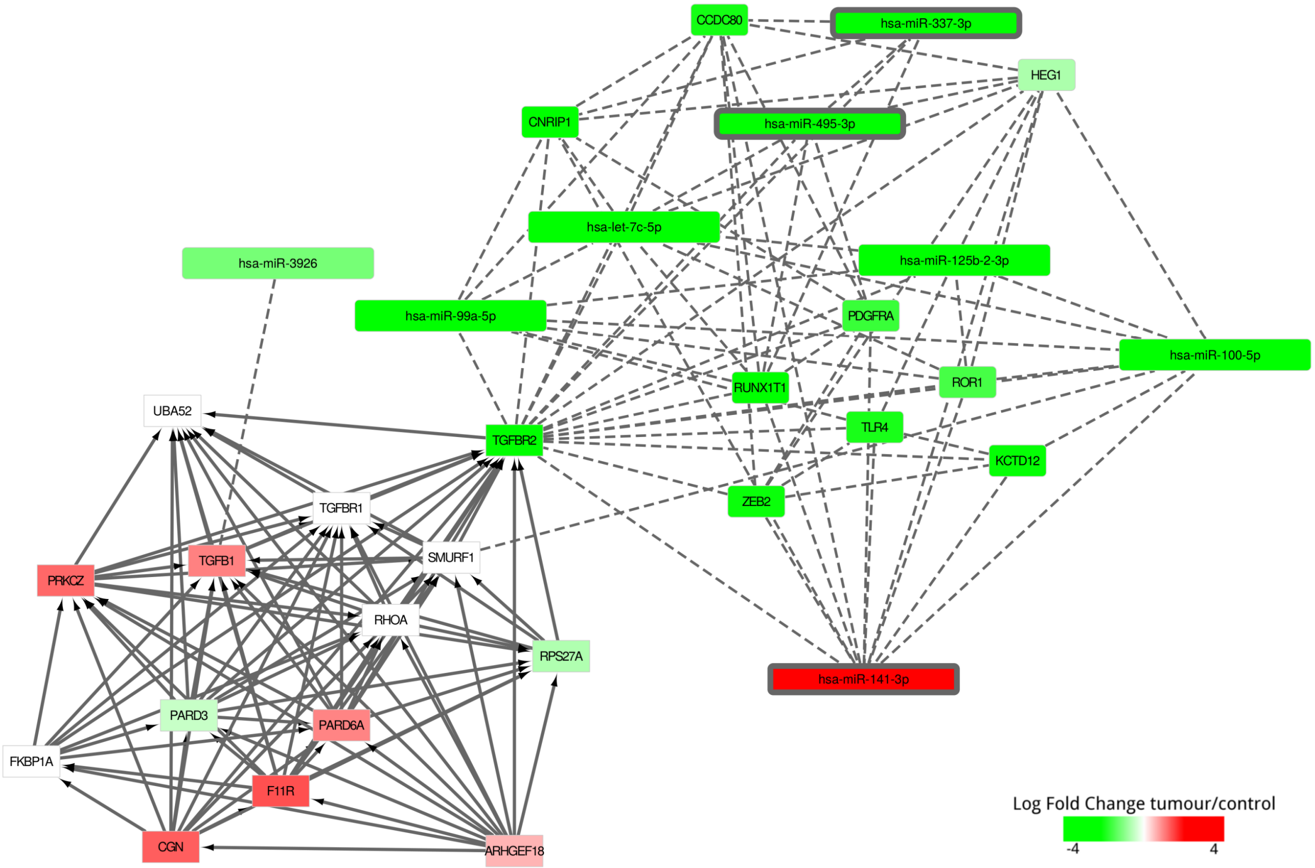

Figure S11. Network visualization of the Reactome pathway: TGF-beta receptor signaling in EMT (epithelial to mesenchymal transition). First neighbours of the nodes in the network inferred from tumour data (miR and mRNA) that matched nodes (genes) in the pathway were added to the visualization. Thick solid stroke edges belong to the pathway; dashed edges correspond to tumour data inferred network associations. miRs from the miR-200 family and the DLK1-DIO3 cluster are highlighted with thicker border.

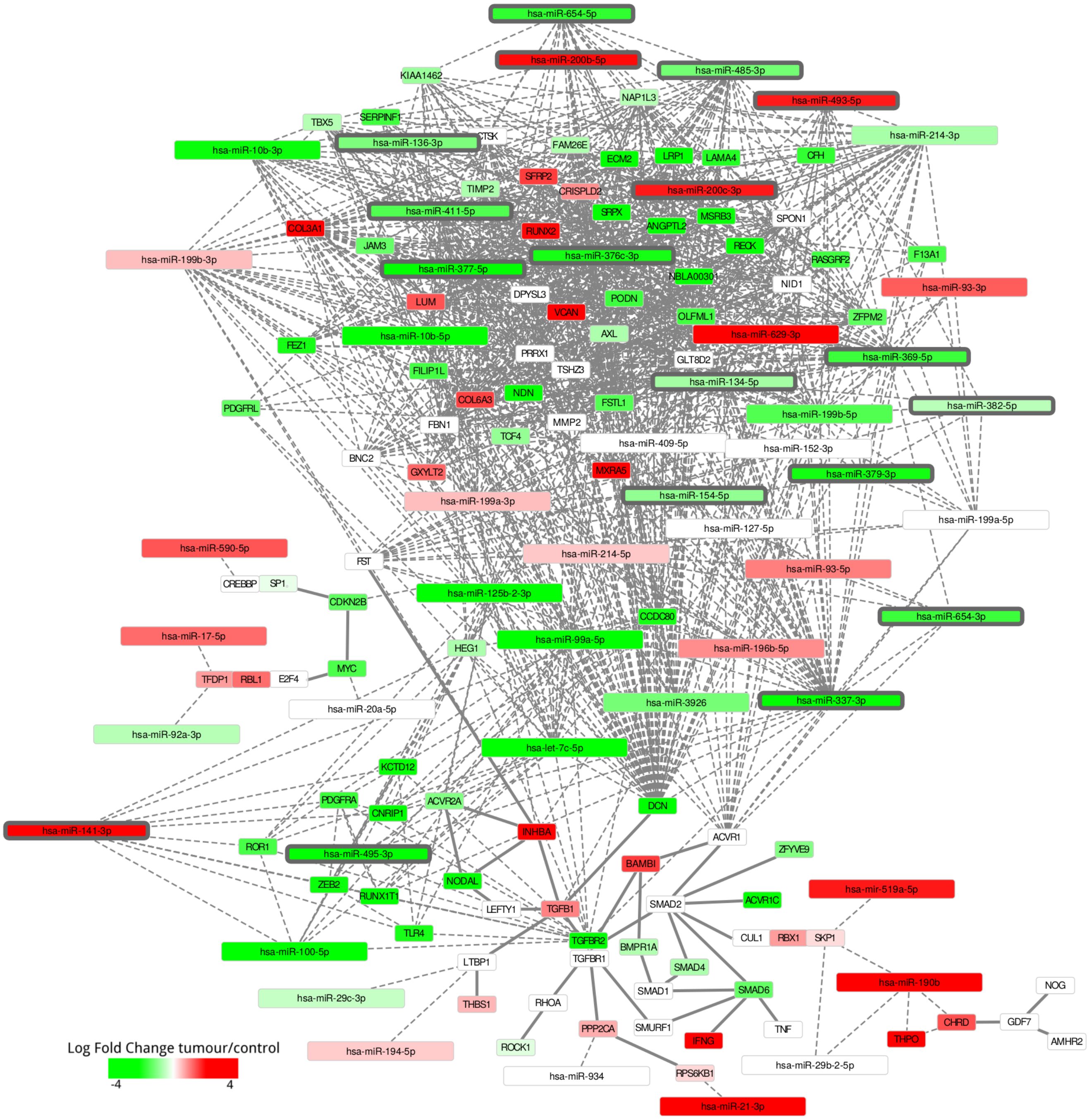

Figure S12. Network visualization of the KEGG pathway: TGF-beta signaling. First neighbours of the nodes in the network inferred from tumour data (miR and mRNA) that matched nodes (genes) in the pathway were added to the visualization. Thick solid stroke edges belong to the pathway; dashed edges correspond to tumour data inferred network associations. miRs from the miR-200 family and the DLK1-DIO3 cluster are highlighted with thicker border.

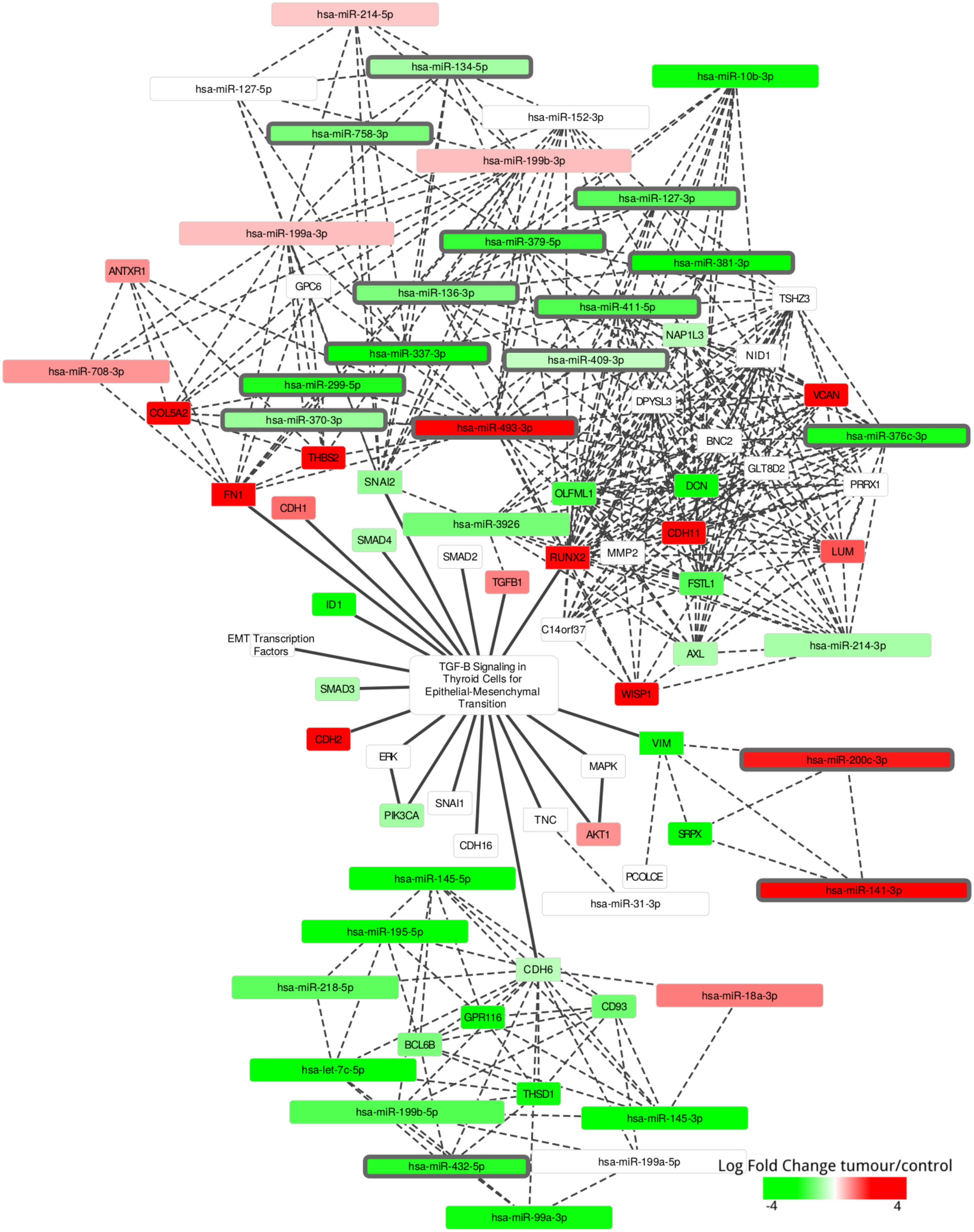

Figure S13. Network visualization of the WikiPathways pathway: TGF-B Signaling in Thyroid Cells for Epithelial-Mesenchymal Transition. First neighbours of the nodes in the network inferred from tumour data (miR and mRNA) that matched nodes (genes) in the pathway were added to the visualization. Thick solid stroke edges belong to the pathway; dashed edges correspond to tumour data inferred network associations. miRs from the miR-200 family and the DLK1-DIO3 cluster are highlighted with thicker border.

Table S17. Information about the node and edge intersection between the networks inferred from the tumour and control data and the experimentally validated interactions from miRTarBase and the miR-target prediction database TargetScan (**Nodes** make reference to the genes that are present in both the inferred networks and the corresponding database. **Nodes w/edges** are the number of genes from **Nodes** with edges in the inferred network that correspond to a predicted or validated interaction). **Common edges** make reference to the edges that are present both at our inferred network and the databases (TargetScan and miRTarBase).

Control				Tumour			
miRTarBase		TargetScan		miRTarBase		TargetScan	
Nodes	2,987	Nodes	2,330	Nodes	3,484	Nodes	2,692
Nodes w/edges	189	Nodes w/edges	239	Nodes w/edges	263	Nodes w/edges	250
Common edges	186	Common edges	298	Common edges	202	Common edges	243

Table S18. Information about the node and edge intersection between the miR-200 first neighbour networks inferred from the tumour and control data and the experimentally validated interactions from miRTarBase and the miR-target prediction database TargetScan (**Nodes** make reference to the genes that are present in both the inferred networks and the corresponding database. **Nodes w/edges** are the number of genes from **Nodes** with edges in the inferred network that correspond to a predicted or validated interaction). **Common edges** make reference to the edges that are present both at our inferred network and the databases (TargetScan and miRTarBase).

Control				Tumour			
miRTarBase		TargetScan		miRTarBase		TargetScan	
Nodes w/edges	48	Nodes w/edges	140	Nodes w/edges	8	Nodes w/edges	30
Common edges	57	Common edges	203	Common edges	5	Common edges	30

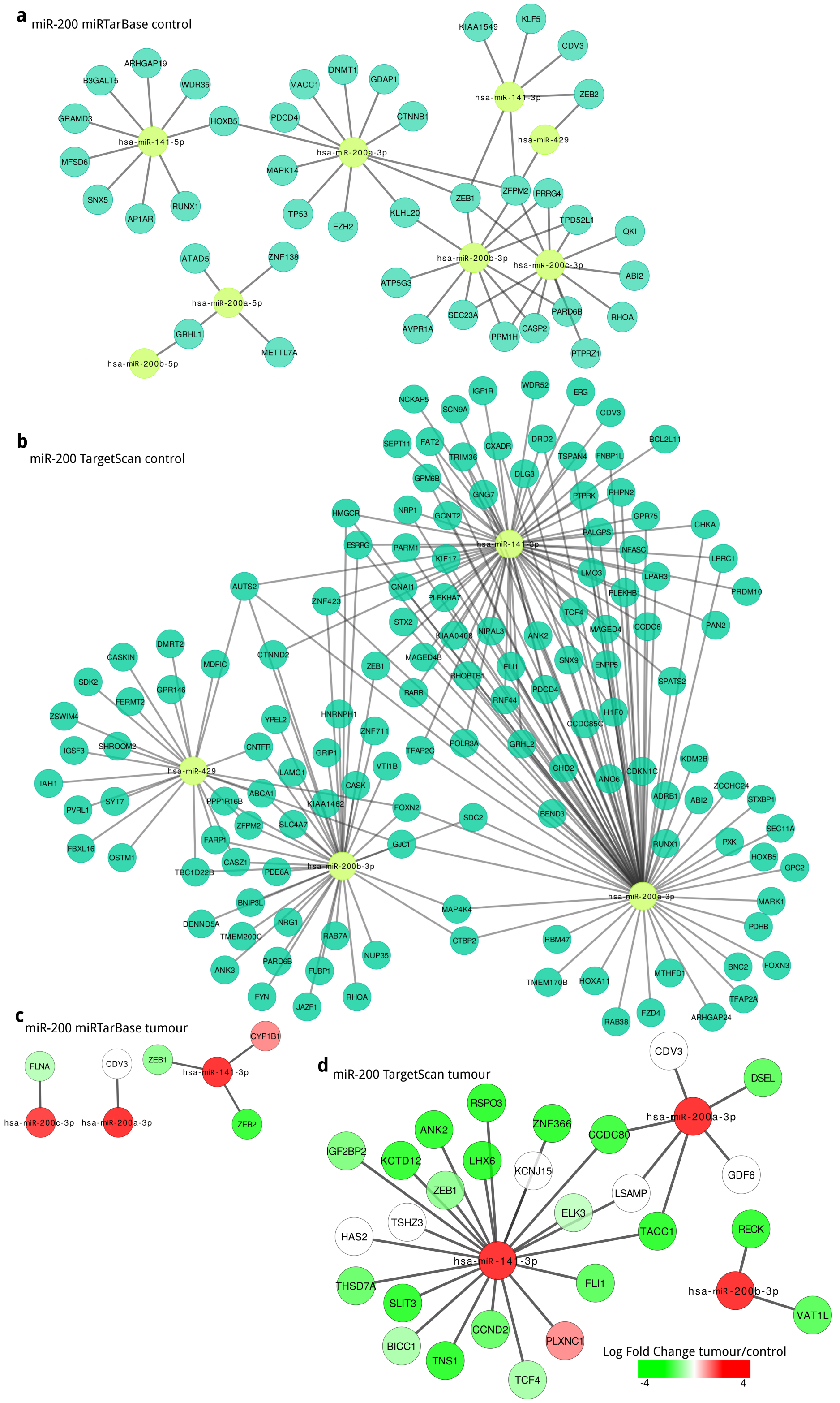

Figure S14. Visualization of the nodes and edges related to miR-200 from the networks inferred from tumour and control samples that match a experimentally validated or predicted miR-target association. The experimentally validated interactions were obtained from miRTarBase, and the predicted miR-target associations were collected from TargetScan.the Tumour networks are coloured by their differential expression.

Supplementary methods

Network construction

We constructed networks with different p-value thresholds to test the susceptibility of the edge inference to this threshold. We found that networks with the same (Supplementary Tables S21 and S22) or different numbers of miR and mRNA edges (at least in two orders of magnitude; Supplementary Tables S23 and S24) have similar network attributes, and disintegration property when miR nodes were removed from the network remains (Supplementary Tables S21-S24). We also determined the top ten degree ranked nodes for each network (Supplementary Tables S25-S28) and found that miR-200 and miR-199 family members remain present as high degree ranked nodes. It is worth noting that even with shifting the thresholds such that the number of edges for miRs an mRNAs are the same, miRs retain their high degree rank.

The p-value thresholds were also modified such that the same number of miR-miR and miR-mRNA edges was the same as the number of mRNA-mRNA edges. These were selected to match the original number of miR-miR and miR-mRNA edges (25,334 edges; i.e. the mRNA-mRNA edges threshold was changed to 99.978%ile; Supplementary Table S21), and the original number of mRNA-mRNA edges (14,892; i.e. the miR-miR and miR-mRNA edge threshold was changed to 99.848%ile; Supplementary Table S22). Also, to test the robustness of our observations to the chosen thresholds, we constructed networks where these these numbers varied by an order of magnitude; both with a decrease (miR edges = 2,533; mRNA edges = 1,489; Supplementary Table S23) and an increase (miR edges = 253,340; mRNA edges = 148,920; Supplementary Table S24). As shown (Supplementary Tables S25-S28), networks with different cut-offs show similar attributes and behaviours to the network reported within the results.

Table S21. Attributes of a variation of the Networks inferred from control and tumour data. The presented networks were constructed adjusting the original mRNA p-value threshold to correspond an initial equal amount of miRs and mRNAs edges (Lc: Largest Component).

Attribute	Whole network		Largest component		Lc mRNA-mRNA subnetwork	
	Control	Tumour	Control	Tumour	Control	Tumour
Total nodes	5,000	4,966	4,624	4,375	4,501	3,888
miR	241	514	123	487	0	0
mRNA	4,759	4,452	4,501	3,888	4,501	3,888
Total interactions	41,572	33,186	41,036	32,747	24,127	16,106
miR-miR	482	1,775	230	1,769	0	0
mRNA-miR	16,777	14,908	16,679	14,872	0	0
mRNA-mRNA	24,313	16,503	24,127	16,106	24,127	16,106
Components	102	241	1	1	1,489	2,325
Single nodes	0	0	0	0	1,449	2,238

Table S22. Attributes of a variation of the Networks inferred from control and tumour data. The presented networks were constructed adjusting the original miR p-value threshold to correspond an initial equal amount of miRs and mRNAs edges (Lc: Largest Component).

Attribute	Whole network		Largest component		Lc mRNA-mRNA subnetwork	
	Control	Tumour	Control	Tumour	Control	Tumour
Total nodes	3,875	3,363	3,501	2,855	3,419	2,461
miR	202	455	82	394	0	0
mRNA	3,673	2,908	3,419	2,461	3,419	2,461
Total interactions	25,905	21,231	25,502	20,874	14,476	10,964
miR-miR	327	1,174	155	1,140	0	0
mRNA-miR	10,941	8,819	10,871	8,770	0	0
mRNA-mRNA	14,637	11,238	14,476	10,964	14,476	10,964
Components	122	204	1	1	1,189	1,379
Single nodes	0	0	0	0	1,157	1,338

Table S23. Attributes of a variation of the Networks inferred from control and tumour data. The presented networks were constructed adjusting the original p-value thresholds that corresponds to a decrease of an order of magnitude of the miRs and mRNAs edge number (Lc: Largest Component).

Attribute	Whole network		Largest component		Lc mRNA-mRNA subnetwork	
	Control	Tumour	Control	Tumour	Control	Tumour
Total nodes	1,096	1,036	823	682	794	610
miR	89	250	29	72	0	0
mRNA	1,007	786	794	610	794	610
Total interactions	3,897	3,566	3,651	3,191	1,315	1,362
miR-miR	103	310	52	158	0	0
mRNA-miR	2,304	1,770	2,284	1,671	0	0
mRNA-mRNA	1,490	1,486	1,315	1,362	1,315	1,362
Components	84	108	1	1	480	380
Single nodes	0	0	0	0	460	372

Table S24. Attributes of a variation of the Networks inferred from control and tumour data. The presented networks were constructed adjusting the original p-value thresholds that corresponds to an increase of an order of magnitude of the miRs and mRNAs edge number (Lc: Largest Component).

Attribute	Whole network		Largest component		Lc mRNA-mRNA subnetwork	
	Control	Tumour	Control	Tumour	Control	Tumour
Total nodes	10,538	15,428	10,439	15,422	9,925	14,789
miR	519	514	514	633	0	0
mRNA	10,019	14,795	9,925	14,789	9,925	14,789
Total interactions	229,249	251,726	229,192	251,723	121,354	65,051
miR-miR	3,588	8,934	3,587	8,934	0	0
mRNA-miR	104,254	177,741	104,251	177,741	0	0
mRNA-mRNA	121,407	65,051	121,354	65,048	121,354	65,048
Components	43	4	1	1	3,646	7,404
Single nodes	0	0	0	0	3,569	7,077

Table S25. Top ten high degree nodes in a a variation of the networks inferred from control and tumour data; constructed adjusting the original mRNA p-value threshold to correspond an initial equal amount of miRs and mRNAs edges.

**Control**		**Tumours**	
Node	Degree	Node	Degree
hsa-mir-200b-3p	1,272	hsa-let-7c-5p	445
hsa-mir-200a-3p	1,247	hsa-mir-199b-5p	435
hsa-mir-141-5p	1,192	hsa-mir-199a-5p	433
hsa-mir-141-3p	1,170	hsa-mir-337-3p	387
hsa-mir-193b-5p	1,025	hsa-mir-99a-5p	366
hsa-mir-200a-5p	1,002	hsa-mir-134-5p	299
hsa-mir-200c-3p	997	hsa-mir-382-5p	293
hsa-mir-22-3p	681	hsa-let-7i-3p	267
hsa-mir-652-3p	640	hsa-mir-199a-3p	262
hsa-mir-200c-5p	625	hsa-mir-199b-3p	260

Table S26. Top ten high degree nodes in a a variation of the networks inferred from control and tumour data; constructed adjusting the original miR p-value threshold to correspond an initial equal amount of miRs and mRNAs edges..

**Control**		**Tumour**	
Node	Degree	Node	Degree
hsa-mir-200a-3p	972	hsa-mir-199b-5p	327
hsa-mir-200b-3p	955	hsa-mir-199a-5p	327
hsa-mir-141-5p	949	hsa-mir-337-3p	319
hsa-mir-141-3p	875	hsa-let-7c-5p	311
hsa-mir-200a-5p	723	hsa-mir-99a-5p	256
hsa-mir-193b-5p	691	hsa-mir-134-5p	238
hsa-mir-200c-3p	641	hsa-mir-199a-3p	234
hsa-mir-22-3p	496	hsa-mir-199b-3p	233
hsa-mir-652-3p	456	hsa-mir-223-3p	214
hsa-mir-378a-5p	399	hsa-mir-150-5p	203

Table S27. Top ten high degree nodes in a a variation of the networks inferred from control and tumour data; constructed adjusting the original p-value thresholds that corresponds to a decrease of an order of magnitude of the miRs and mRNAs edge number.

**Control**		**Tumour**	
Node	Degree	Node	Degree
hsa-mir-200a-3p	231	hsa-mir-150-5p	181
hsa-mir-224-5p	215	hsa-mir-142-5p	145
hsa-mir-652-3p	213	hsa-mir-155-5p	126
hsa-mir-141-5p	198	hsa-mir-199a-3p	111
hsa-mir-200b-3p	197	hsa-mir-199b-3p	110
hsa-mir-141-3p	191	hsa-mir-146a-5p	103
hsa-mir-452-5p	160	hsa-mir-134-5p	99
hsa-mir-378a-5p	148	hsa-mir-199b-5p	95
hsa-mir-200a-5p	120	hsa-mir-199a-5p	93
hsa-mir-224-3p	86	hsa-mir-337-3p	87

Table S28. Top ten high degree nodes in a a variation of the networks inferred from control and tumour data; constructed adjusting the original p-value thresholds that corresponds to an increase of an order of magnitude of the miRs and mRNAs edge number.

**Control**		**Tumour**	
Node	Degree	Node	Degree
hsa-mir-193b-5p	2,563	hsa-mir-190b	1,771
hsa-mir-200a-3p	2,469	hsa-mir-199a-5p	1,668
hsa-mir-200b-3p	2,408	hsa-mir-29b-2-5p	1,590
hsa-mir-141-5p	2,379	hsa-mir-199b-5p	1,475
hsa-mir-200c-3p	2,348	hsa-mir-337-3p	1,392
hsa-mir-141-3p	2,280	hsa-mir-18a-3p	1,370
hsa-mir-200a-5p	2,260	hsa-mir-452-3p	1,306
hsa-mir-200c-5p	2,,193	hsa-mir-382-5p	1,246
hsa-mir-652-3p	2,014	hsa-mir-18a-5p	1,228
hsa-mir-146b-5p	1,984	hsa-mir-29c-5p	1,173
